# Ferroptosis and immunity: a bibliometric analysis of research hotspots and frontiers (2012-2025)

**DOI:** 10.3389/fimmu.2025.1739210

**Published:** 2026-01-12

**Authors:** Qi Huang, Shuhang Feng, Changhao He, Rong Xu, Yixuan Cai, Linyu Guan, Jiaqi Guan, Lei Wang

**Affiliations:** 1 School of Pharmaceutical Sciences, Zhejiang Chinese Medical University, Hangzhou, China; 2 School of Public Health, Zhejiang Chinese Medical University, Hangzhou, China

**Keywords:** bibliometrics, cancer immunotherapy, ferroptosis, immunity, tumor immune microenvironment

## Abstract

**Background:**

Ferroptosis is a form of programmed cell death triggered by iron-dependent lipid peroxidation. Accumulating evidence has revealed potential associations between cellular Ferroptosis and infection, inflammation, and immunity. Given the substantial literature in this field, the present study aims to comprehensively overview the research hotspots and knowledge structure underlying the interplay between Ferroptosis and immunity via bibliometric analysis.

**Methods:**

Relevant literature focusing on Ferroptosis and immunity—published between January 1, 2012, and August 31, 2025—was retrieved from the Web of Science, PubMed, and Scopus databases. Bibliometric analyses, encompassing evaluations of national contributions, institutional collaborative networks, journal outputs, and keyword co-occurrence, were conducted using CiteSpace, VOSviewer, and Origin software. These analyses aimed to delineate research trends and project future research trajectories.

**Results:**

A total of 3,436 publications relevant to Ferroptosis and immunity were included in this study. The analysis revealed a continuous and steady growth in the number of articles published in this field over the past 12 years. At the national level, China leads in both publication output (H-index = 99) and institutional contributions, with Central South University and Sun Yat-sen University each accounting for 154 papers. In contrast, the United States (average citations per paper: 114.11) and Germany (average citations per paper: 142.92) demonstrate outstanding performance in research impact. Frontiers in Immunology stands out as the most prolific journal, with 162 published articles in this field. Keyword co-occurrence analysis indicated that the application of Ferroptosis in cancer immunotherapy currently constitutes the most prominent research focus.

**Conclusion:**

This study represents the first systematic bibliometric analysis of literature on Ferroptosis and immunity, leveraging data from the Web of Science, PubMed, and Scopus databases. These findings underscore that oncology and immunology are the dominant disciplines in this field, with Ferroptosis-targeted cancer immunotherapies and the modulation of the tumor immune microenvironment (TME) emerging as key frontiers. Strengthening international collaboration and focusing on high-impact research will facilitate the clinical translation of therapeutic strategies for immunity-related diseases.

## 1 Introduction

Ferroptosis, a distinct form of iron-dependent regulated cell death triggered by iron overload and lipid peroxidation (1), has emerged as a rapidly growing research focus across multiple disciplines since its first identification in 2012. This unique cell death modality is tightly regulated by a spectrum of cellular metabolic processes, including redox homeostasis, iron metabolism, mitochondrial activity, and the metabolism of amino acids, lipids, and carbohydrates. Additionally, it is modulated by a numerous disease-associated signaling pathways (2). Notably, Ferroptosis servers as a key driver of organ damage and degenerative pathologies in numerous clinical contexts. For instance, it has been implicated in diverse pathological conditions such as cancer, eurodegenerative diseases, tissue damage, infection, inflammation, and immunity, all of which exhibit close associations with the human immune system (3). The crosstalk between cellular Ferroptosis and infection, inflammation, as well as the immune system is rooted in their profound interactions at the levels of oxidative stress and metabolism. Emerging evidence has demonstrated that pathogen infection can directly induce mitochondrial oxidative stress and iron metabolism dysregulation, thereby triggering the ferroptotic program in host cells (4). Concomitantly, the release of alarm signals such as damage-associated molecular patterns (DAMPs) during this process potently activates the innate immune response (5). In the context of inflammation, classical pro-inflammatory cytokines modulate cellular susceptibility to Ferroptosis, while lipid peroxidation products generated by Ferroptosis act as potent pro-inflammatory mediators per se, thus forming a self-amplifying pathological cycle (6).

The immune system serves as the body’s core defense against pathogen invasion and a key regulator of internal homeostasis, orchestrating the recognition and elimination of abnormal cells through the synergistic coordination of innate and adaptive immunity. Emerging evidence over recent years has underscored that Ferroptosis plays a pivotal role in regulating the function of innate immune cells (7). Notably, this regulation operates bidirectionally: On one hand, the immune system can precisely modulate Ferroptosis progression by secreting cytokines, chemokines, and metabolites. On the other hand, conversely, ferroptotic cells release a spectrum of signaling molecules—including Damage-Associated Molecular Patterns (DAMPs)—which are readily detected by immune cells; this detection, in turn, modulates immune cell differentiation, activation, migration, and other biological behaviors, thereby reshaping the immune microenvironment (8). These two interconnected and synergistic processes play a critical role in driving the pathogenesis and progression of diverse diseases, such as cancer, neurodegenerative diseases, cardiovascular diseases, and autoimmune disorders (9, 10). Elucidating the intrinsic crosstalk between Ferroptosis and immunity is essential for deepening our understanding of disease pathogenesis. Targeting these interaction nodes could therefore enable effective disease intervention, offering new prospects for improving patient prognosis (11). Consequently, in-depth investigation into the Ferroptosis-immunity axis carries substantial theoretical value and broad clinical translational potential.

Since its discovery in 2012, the volume of publications on Ferroptosis research has expanded exponentially over the past decade. By clarifying the regulatory mechanisms governing Ferroptosis and its associations with diverse diseases, Ferroptosis has emerged as a highly promising therapeutic target (12). However, the rapid advancement of this research field has also brought new challenges—particularly limitations in experimental methodologies—that may lead to misinterpretation of Ferroptosis assessment. Thus, synthesizing recent literature on the interplay between Ferroptosis and immunity is essential, with the goal of offering practical recommendations to guide future in-depth investigations in this field.

Distinct from traditional narrative reviews, bibliometric analysis utilizes statistical methodologies to quantitatively analyze scientific literature. It identifies leading institutions/countries, core authors, key journals, and highly cited references, while also characterizing research trends or hotspots. Furthermore, it facilitates the accurate identification of research foci and exploration of novel research directions via software-assisted visual analysis of literature—including tools such as CiteSpace, VOSviewer, and Origin (13). Given the complexity and vastness of the existing literature on the Ferroptosis-immunity axis, traditional narrative reviews may not be sufficient to comprehensively grasp the research landscape. Therefore, this study employs a bibliometric framework to conduct a visual analysis of relevant literature, aiming to provide a more systematic and in-depth understanding of their interplay. This approach can not only identify key research areas and emerging trends but also offer valuable insights for future research directions.

## 2 Materials and methods

### 2.1 Data sources

This study retrieved literature on Ferroptosis and immunity from three databases: Web of Science Core Collection (WoSCC), PubMed, and Scopus. All searches were conducted on the same day to avoid potential bias introduced by daily database updates.

### 2.2 Search strategy

The search strategy employed in the WoS database was formulated as follows: TS=((Ferroptosis) AND (Immune OR Immunity)). The search was restricted to English-language articles and reviews published between January 1, 2012, and August 31, 2025.

For the PubMed database, the search query was defined as: ((Immune[Title/Abstract]) OR (Immunity[Title/Abstract])) AND (Ferroptosis[Title/Abstract]). The following filters were applied to refine the search results: document type (Article or Review), language (English), and publication date (January 1, 2012, to August 31, 2025).

In the Scopus database, the search formula used was: (TITLE-ABS-KEY (Ferroptosis) AND TITLE-ABS-KEY (Immune OR Immunity)). The retrieval was limited to English-language Articles and Reviews, with the publication time frame spanning from 2012 to 2025.

### 2.3 Data screening

#### 2.3.1 Inclusion criteria

Literature related to Ferroptosis and immunity was retrieved, and the assessment of literature relevance was conducted via independent review by two researchers. The review criterion was that both Ferroptosis-associated terms (e.g., Ferroptosis) and immunity-associated terms (e.g., Immune, Immunity) were present in the title or abstract. A total of 4451 publications were obtained through this retrieval process.

#### 2.3.2 Exclusion criteria

**Figure 1:**
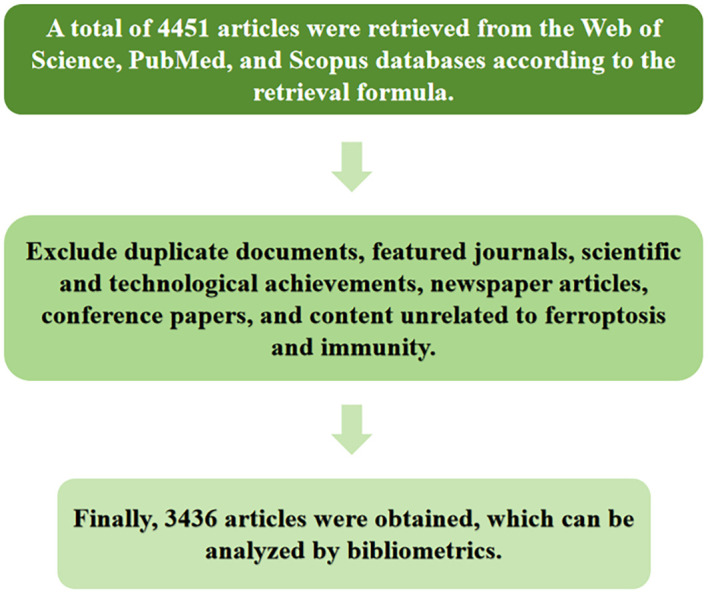
Literature screening flowchart.

(1) Duplicate records; (2) Featured journals, scientific and technological achievements, newspaper articles, and conference proceedings; (3) Publications unrelated to Ferroptosis and immunity. A total of 3,436 publications ultimately met the inclusion criteria. The search results are illustrated in **Figure 1**.

### 2.4 Data visualization

Retrieve the relevant literature and export it in both *RefWorks* and plain text formats, naming the files as “download-0*.txt”. Utilize CiteSpace 6.4.R1 to perform data deduplication and format conversion. Configure the following parameters: (1) Time slicing: January 1, 2012 – August 31, 2025; set the slice length to “1”. (2) Node types: Select “Author”, “Institution”, and “Keyword” for analysis. (3) Selection criteria: Configure g-index (K = 25), Top N = 50, and Top N%=10.0%. (4) Pruning method: Apply “Pathfinder” and “Pruning sliced network” algorithms. Conduct data preprocessing followed by visual analytics to investigate publication trends, institutional co-occurrence, research collaboration patterns, keyword clustering hotspots, and disciplinary orientations. Generate foundational statistical tables using Excel to enhance data interpretability. Employ VOSviewer to analyze keyword co-occurrence networks based on the exported *RefWorks* files.

## 3 Results

### 3.1 Publication trends

**Figure 2:**
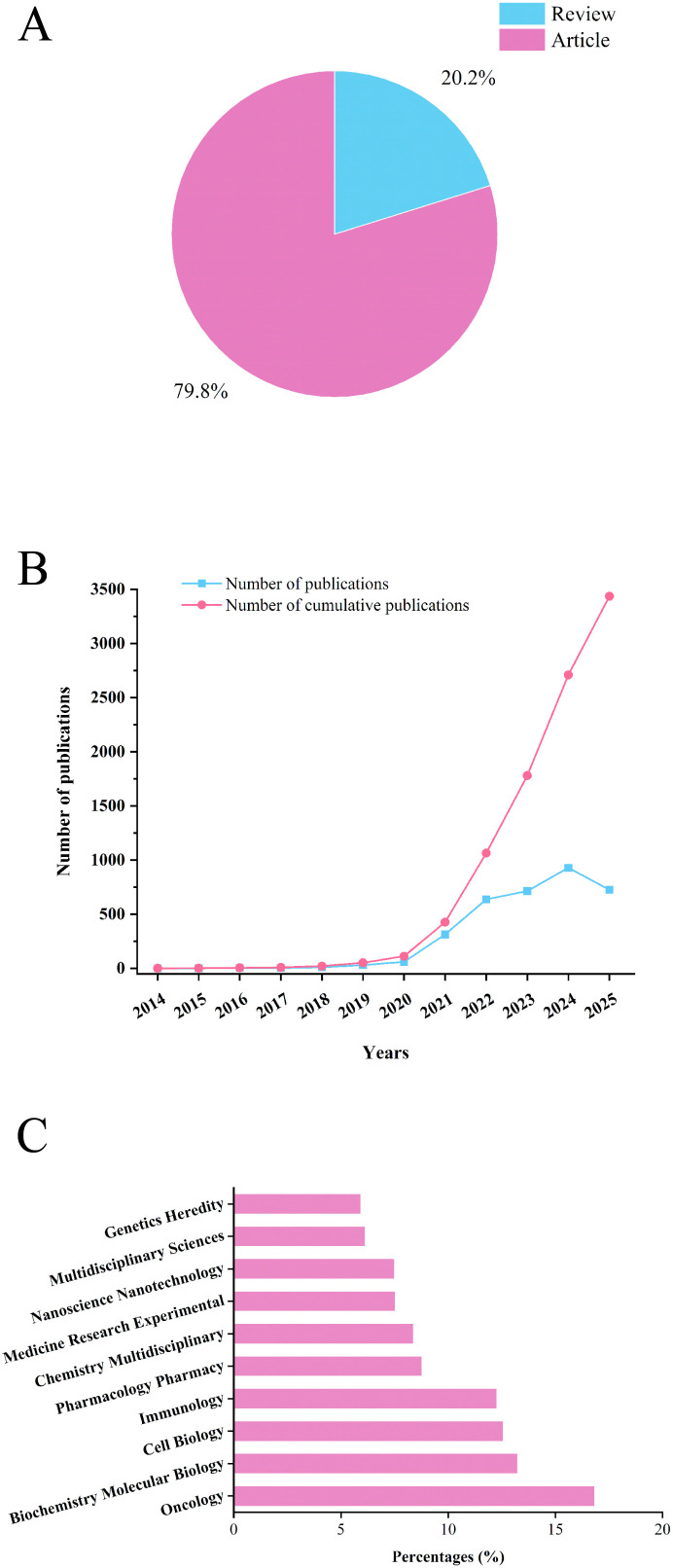
Number and type of literature related to ferroptosis and immunity. **(A)** Literature type distribution. **(B)** Annual publications quantitative distribution. **(C)** Subject categories distribution.

To characterize publication trends in Ferroptosis-immunity research, this study retrieved relevant literature (published January 1, 2012–August 31, 2025) from the WoSCC, PubMed, and Scopus databases via subject term-based searches. Following the exclusion of irrelevant articles per predefined criteria, a total of 3,436 publications were included—consisting of 2,742 research articles and 694 review articles (**Figure 2A**). Analysis of these trends indicates that Ferroptosis-immunity research had already garnered initial attention prior to 2020. Early studies had established that Ferroptosis occurs in tumor cells and contributes to cancer immunity (14), laying a foundational framework for subsequent investigations. From 2021 to 2025, the annual number of publications increased markedly, solidifying Ferroptosis-immunity as a globally prominent research focus. Two key factors likely drove this sharp growth: first, the widespread adoption of cutting-edge technologies—including gene editing (e.g., CRISPR-Cas9), single-cell sequencing, and nanotechnology—in Ferroptosis-immunity research (15–17); second, significant advances in elucidating the roles of Ferroptosis in tumor immunotherapy and autoimmune diseases (18). These developments have collectively established Ferroptosis-immunity as a research hotspot in the medical field (**Figure 2B**). Looking forward, as Ferroptosis-immunity research advances—coupled with expanded research funding support and strengthened academic exchanges—the number of publications is projected to keep growing over the next decade. Importantly, this progress may also facilitate the translation of key theoretical findings into clinical practice. For the subject categories of the included literature (**Figure 2C**), the top five fields were as follows: Oncology (578 publications, 16.8%), Biochemistry Molecular Biology (454 articles, 13.2%), Cell Biology (431 articles, 12.5%), Immunology (421 articles, 12.3%), and Pharmacology Pharmacy (301 articles, 8.8%).

### 3.2 Analysis of countries, institutions, and journals

#### 3.2.1 Geographical distribution map of countries and regions

**Figure 3:**
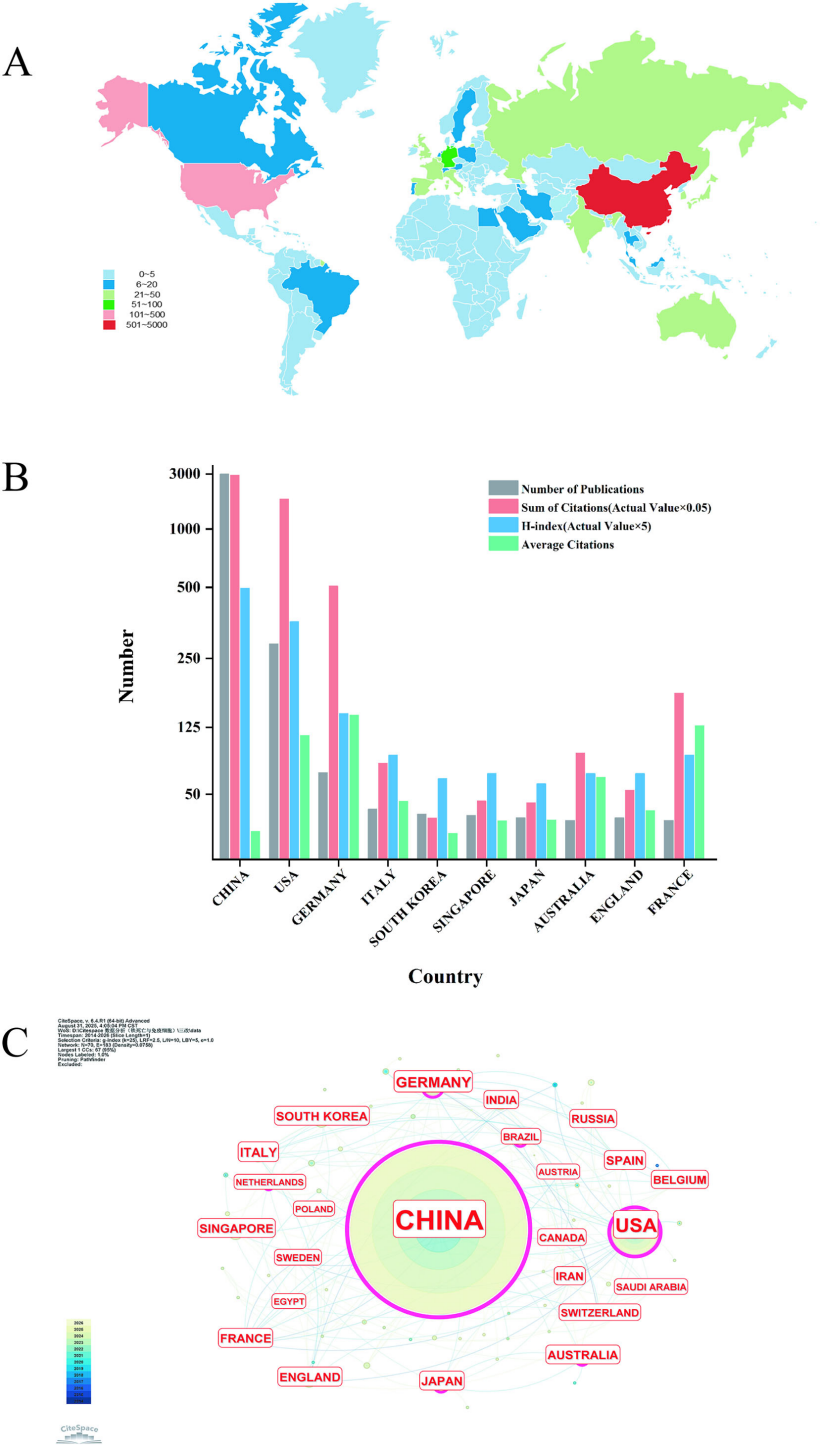
Global contribution map of scientific publications. **(A)** Overall situation of number of publications worldwide. **(B)** Number of publications, sum of citations (×0.05), H-index (×5), and average citations in the top 10 countries or regions. **(C)** International Collaborative Publication Relationships among Countries.

Globally, research on Ferroptosis and immunity is being conducted across 70 countries and regions, with their respective publication contributions visualized in **Figure 3A**. China leads with the highest number of publications—its output far surpassing the combined total of all other countries and regions. Beyond China, the United States, Germany, Italy, and South Korea have also produced substantial research output in this field. Notably, China also ranks first in the overall H-index (99) of publications in this domain. However, it lags behind other leading countries in average citations per article (19.52), a trend that suggests room for improvement in the impact of Chinese publications. The United States performs strongly in both metrics: it ranks second in overall H-index (71) and second in average citations per article (114.11), reflecting a balanced strength in both research quantity and publication influence. Germany presents a distinct pattern: despite a lower overall publication volume, it ranks first in average citations per article (142.92), highlighting the high impact of its research in this field (**Figure 3B**). China leads in the number of publications, which may be attributed to the inclusion of “tumor immunity and cell death regulation” as a key research priority in China’s “14th Five-Year Plan”. In contrast, the average citation frequency of papers from the United States is higher than that from China, which is presumably due to two factors: first, U.S. research focuses more on original innovative mechanisms; second, a larger proportion of U.S. studies are published in high-impact journals.

**Figure 3C** presents a network visualization of international collaborative publications derived from the WOS database. Within this figure, node size corresponds to the volume of publications, while node color encodes the chronological sequence of publication. Analysis parameters (top-left corner) reveal 70 nodes and 183 edges in total—an observation that reflects intensive international research collaboration in this field. Notably, the connecting edges are predominantly yellow, green, and blue; this color distribution indicates that cross-country collaborative publications over the past five years have been highly frequent, constituting the overwhelming majority of total collaborative publications. Looking ahead, the volume of literature focusing on Ferroptosis and immunity is anticipated to keep expanding over the next 5–10 years, with international collaborative efforts becoming increasingly intensive.

#### 3.2.2 Institutional collaboration network map

**Figure 4:**
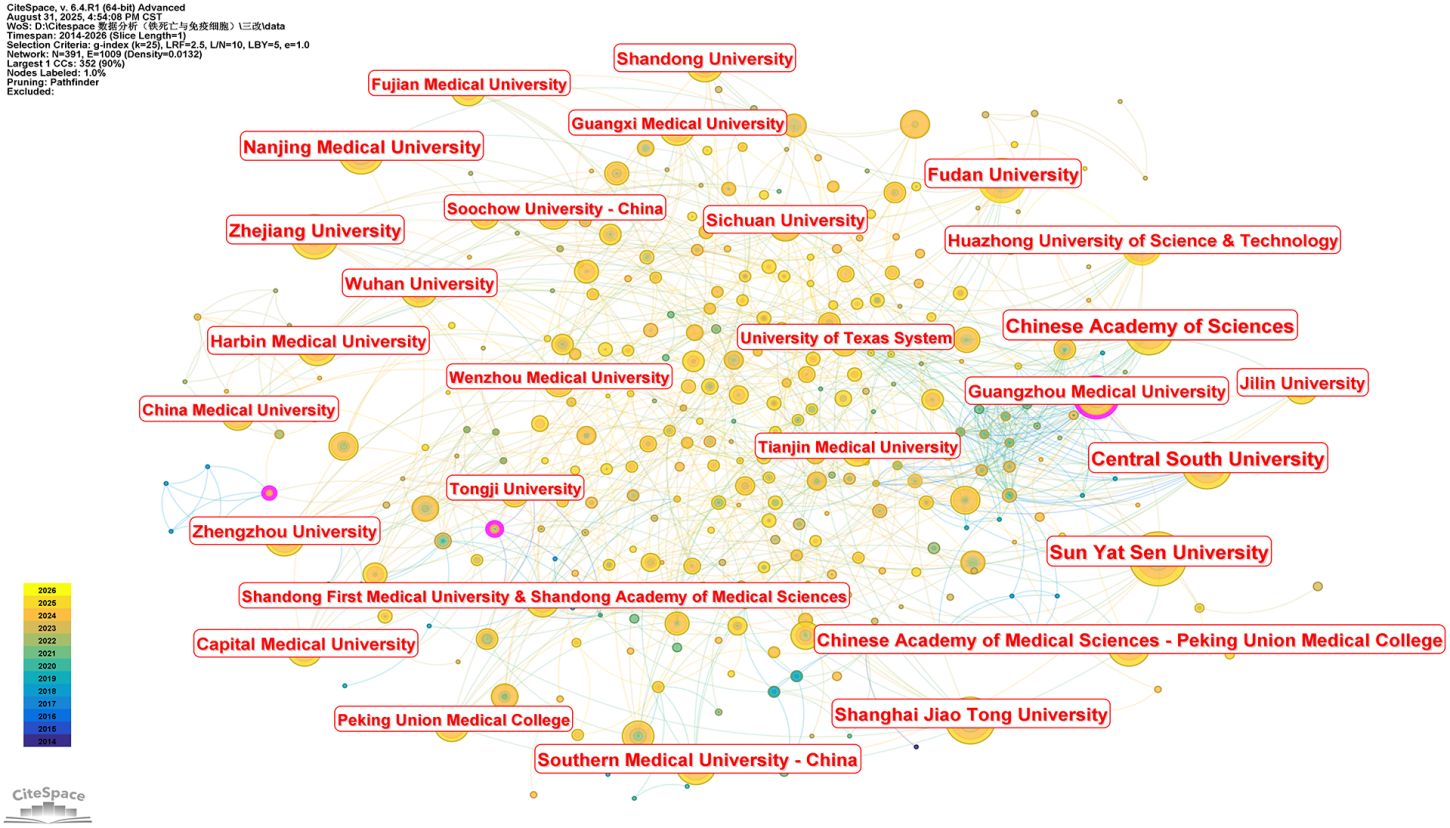
Collaboration network diagram of research institutions in the literature.

The collaborative network of research institutions, constructed based on publication data, is presented in **Figure 4**, which includes 391 nodes and 1,009 edges. Across the three databases, the institutions ranked top in publication output are Central South University and Sun Yat-sen University, each with 154 publications. They are followed by Fudan University and Shanghai Jiao Tong University, both recording 123 publications, and then Zhejiang University, with 116 publications. Analysis of this institutional collaboration network further indicates that the top three institutions in terms of publication volume are all Chinese higher education institutions—and these leading entities exhibit extensive inter-organizational collaborative ties. This observation underscores the growing integration of Chinese higher education institutions into the global academic community, as evidenced by the active publication of research findings by numerous domestic universities in international journals and platforms. This trend reflects the advancing internationalization of China’s higher education system and the expanding global academic impact of its institutions.

#### 3.2.3 Author publication contribution map

**Figure 5:**
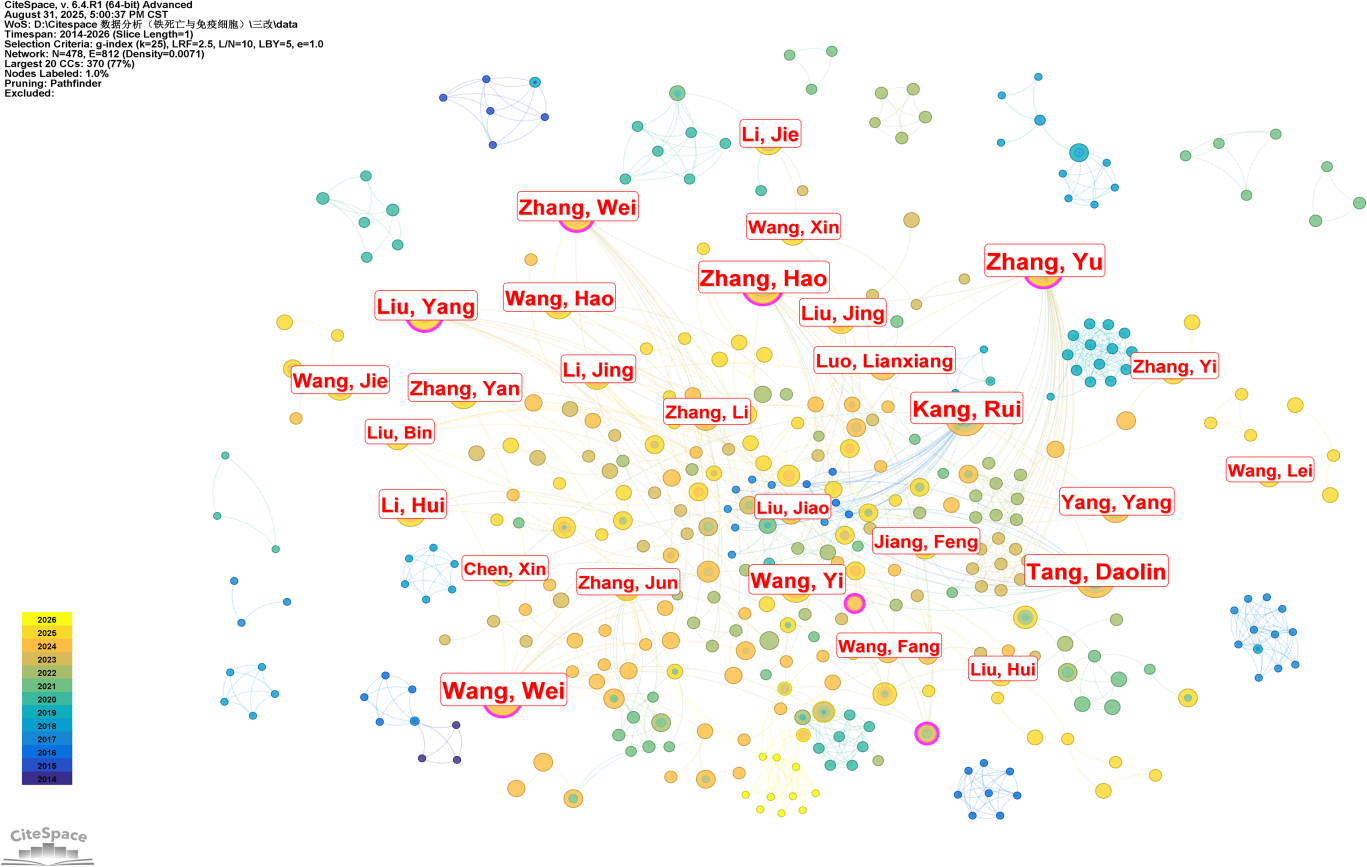
Author contributions figure. Node size represents the number of articles published by the author, while the connections represent collaborative relationships between authors.

An analysis of the retrieved literature demonstrates that a total of 19,846 authors have contributed to Ferroptosis-immunity research. **Figure 5** and **Table 1** summarize the most prolific authors in this field. Notably, Kang, Rui emerges as the most productive contributor, with 29 publications over the past 13 years, followed by Tang, Daolin and Zhang, Yu (each with 28 publications) and Wang, Wei (27 publications). Notably, however, the number of publications alone is not a reliable metric for evaluating research output—citation counts better reflect an author’s academic influence (19). Among the top 10 authors listed in **Table 1**, seven have an average number of citations per publication surpassing 500. Furthermore, Tang, Daolin has the highest total citations (5,286) across his publications, indicating that his work has exerted a profound impact on Ferroptosis-immunity research.

**Table 1:** Top 10 authors by publication volume.

Rank	Author	Documents	Citations
1	Kang, Rui	29	5170
2	Tang, Daolin	28	5286
3	Zhang, Yu	28	746
4	Wang, Wei	27	879
5	Zhang, Wei	22	419
6	Zhang, Hao	22	335
7	Liu, Yang	21	680
8	Wang, Yi	21	266
9	Yang, Yang	19	410
10	Li, Hui	19	240

#### 3.2.4 Journal publication contribution map

**Figure 6:**
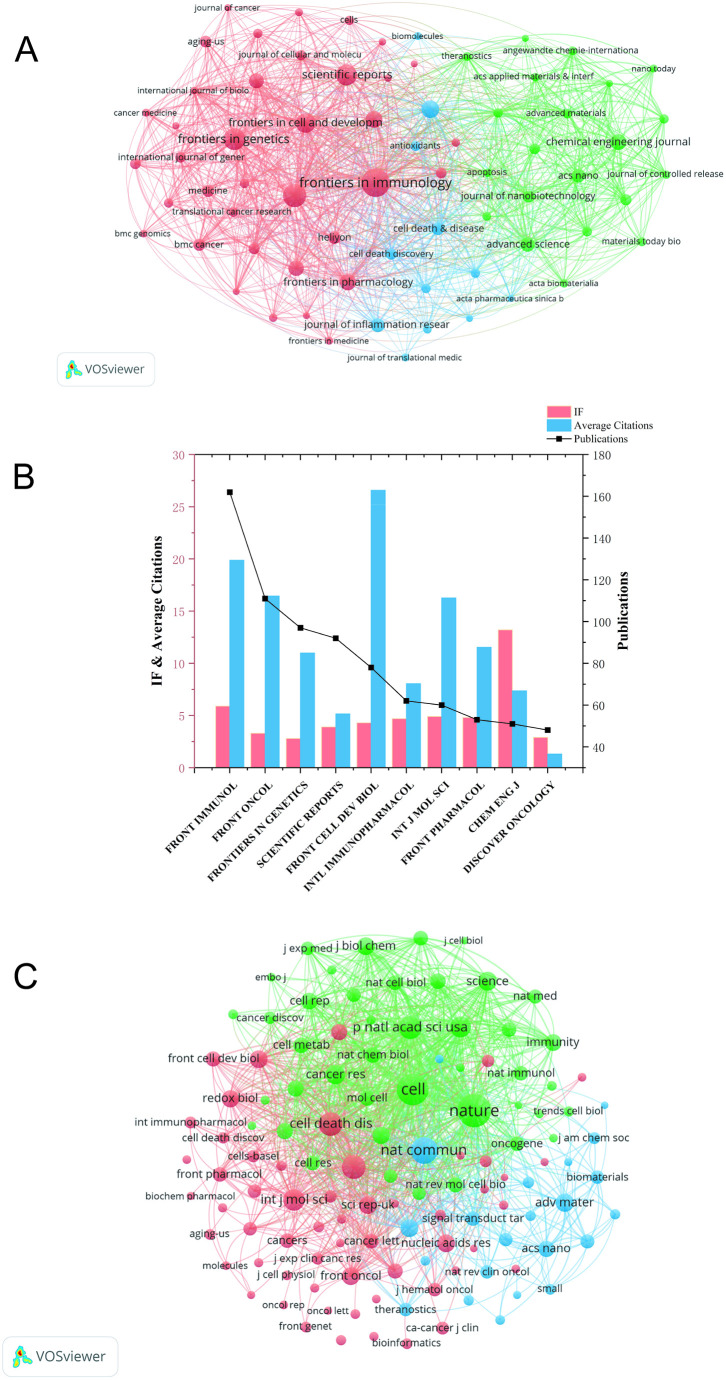
Journal publication contribution map. **(A)** Publication distribution of Ferroptosis and immunity research across journals. Circle size corresponds to the publication count per journal. **(B)** Number of publications, Impact Factor, and average citations in the top 10 journals. **(C)** Journal co-citation network. Circle size indicates stronger co-citation relationships between journals.

**Figure 6A** illustrates the journal publication network, which comprises three distinct clusters—evidenced by the three distinct colors in the visualization. The top three journals ranked by publication output are detailed in **Figure 6B**: Frontiers in Immunology (162 articles, impact factor: 5.9, average citations per paper: 19.88), Frontiers in Oncology (111 articles, impact factor: 3.3, average citations per paper: 16.45), and Frontiers in Genetics (97 articles, impact factor: 2.8, average citations per paper: 10.99). Frontiers in Immunology has the highest publication output (162 articles), which is highly aligned with its journal positioning of “focusing on immunology-related interdisciplinary research.” Notably, the “Cell Death and Immunity” Research Topic launched by this journal has further attracted high-quality submissions in the relevant field.

As depicted in **Figure 6C**, the journal co-citation network is delineated into three distinct clusters, with each cluster corresponding to one of the three color-coded groups in the visualization. Among these, the three most highly cited journals are Nature (with 7,059 citations), Cell (with 6,407 citations), and Nature Communications (with 4,678 citations). Notably, all three journals fall within the Q1 category of the Journal Citation Reports (JCR), as classified by the Institute for Scientific Information (ISI).

#### 3.2.5 Analysis of top-cited literature

Citation frequency serves as a pivotal metric for gauging current research dynamics and predicting future trends. **Table 2** enumerates the top 10 most highly cited articles across the three databases. The most highly cited publication—”Ferroptosis: mechanisms, biology and role in disease” (with 4,698 citations), published in Nature Reviews Molecular Cell Biology—offers a comprehensive and critical overview of Ferroptosis, including its molecular mechanisms, regulatory networks, potential physiological roles in tumor suppression and immune surveillance, as well as its pathological implications and therapeutic prospects (2). The second most highly cited article—”Ferroptosis: process and function” (with 2,854 citations), published in Cell Death and Differentiation—systematically summarizes the regulatory mechanisms and signaling pathways of Ferroptosis, while also discussing its pathophysiological significance in human diseases (10).

**Table 2:** Top 10 most cited publications.

Rank	Article title	Author	Journal	Years	Cited quantity
1	Ferroptosis: mechanisms, biology and role in disease	Jiang, Xuejun	NATURE REVIEWS MOLECULAR CELL BIOLOGY	2021	4698
2	Ferroptosis: process and function	Xie, Y	CELL DEATH AND DIFFERENTIATION	2016	2845
3	CD8+ T cells regulate tumour ferroptosis during cancer immunotherapy	Wang, Weimin	NATURE	2019	2070
4	Ferroptosis turns 10: Emerging mechanisms, physiological functions, and therapeutic applications	Stockwell, BR	CELL	2022	1808
5	Targeting ferroptosis as a vulnerability in cancer	Lei, Guang	NATURE REVIEWS CANCER	2022	1545
6	NLRP3 inflammasome activation and cell death	Huang, Yi	CELLULAR & MOLECULAR IMMUNOLOGY	2021	976
7	Ferroptosis at the crossroads of cancer-acquired drug resistance and immune evasion	Angeli, JPF	NATURE REVIEWS CANCER	2019	951
8	Ferroptosis, necroptosis, and pyroptosis in anticancer immunity	Tang, R	JOURNAL OF HEMATOLOGY & ONCOLOGY	2020	921
9	Radiotherapy and Immunotherapy Promote Tumoral Lipid Oxidation and Ferroptosis via Synergistic Repression of SLC7A11	Lang, Xueting	CANCER DISCOVERY	2019	789
10	Ferroptosis at the intersection of lipid metabolism and cellular signaling	Liang, Deguang	MOLECULAR CELL	2022	746

### 3.3 Keywords analysis

**Figure 7:**
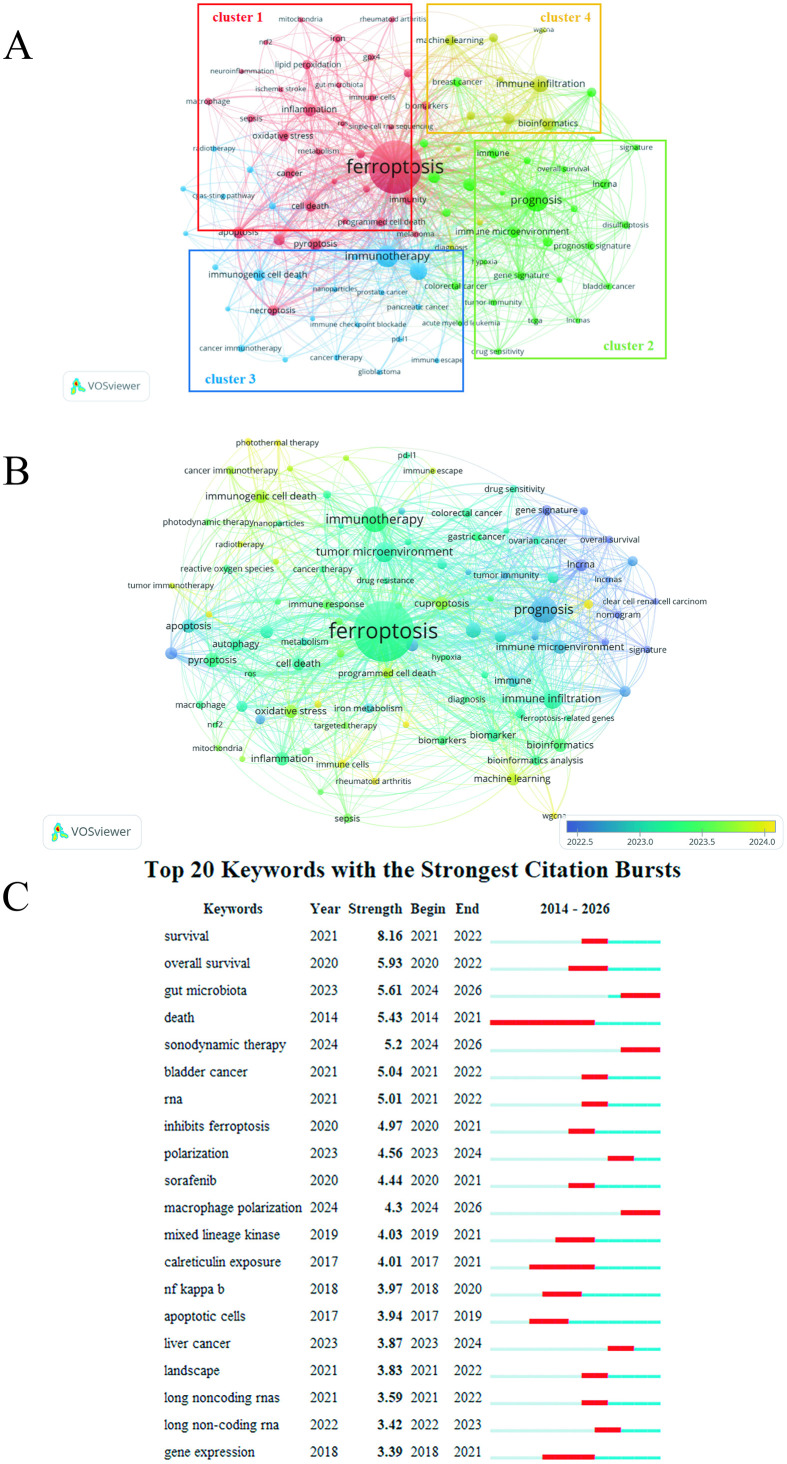
Keyword analysis map. **(A)** Identification of Key Terms in the Field of Ferroptosis and Immunology. The size of the circle represents the frequency of keyword occurrence, and different colors represent different clusters. **(B)** Distribution of keywords according to the average time of appearance. Blue represents an early appearance, and yellow represents a late appearance. **(C)** Top 20 most cited keywords receiving consistent attention over the period. Red bars represent keywords frequently cited during this timeframe.

Keywords represent the essence of literature content. Through analytical examination, they can effectively reveal the core intentions of research articles and help identify critical domains and exploration directions in scientific investigations. A visual co-occurrence analysis of the literature on Ferroptosis and immunity in the three databases was performed, and the results were shown in **Supplementary Figure 1**. The top 30 high-frequency keywords are shown in **Supplementary Table 1**. We classified 97 keywords with frequency ≥15 occurrences from 3,436 publications. The keywords in VOSviewer are divided into 14 clusters (**Supplementary Figure 2**), while the results of CiteSpace are divided into four clusters, each of which represents different aspects of the research field. As shown in **Figure 7A**: the red cluster (Cluster 1) is associated with the core mechanisms of Ferroptosis and underlying diseases, with keywords including Ferroptosis (1,967 occurrences), pyroptosis (114), inflammation (102), apoptosis (100), cell death (92), and oxidative stress (92). The green cluster (Cluster 2) relates to prognostic modeling and biomarker mining of Ferroptosis in cancer, featuring keywords such as prognosis (362), hepatocellular carcinoma (123), cuproptosis (100), immune microenvironment (100), immunity (75), prognostic model (69), and lung adenocarcinoma (66). The blue cluster (Cluster 3) is linked to Ferroptosis-targeted cancer therapeutic strategies, including immunotherapy (344), immunogenic cell death (101), tumor immune microenvironment (51), cancer immunotherapy (42), and chemotherapy (35). The yellow cluster (Cluster 4) involves bioinformatic analysis, genetic signatures, and machine learning, with keywords including immune infiltration (191), bioinformatics (102), biomarker (88), machine learning (84), and bioinformatic analysis (58). Beyond analyzing keywords within these domains, we evaluated the recent research attention received by each cluster. **Figure 7B** demonstrates that Cluster 3 currently receives comparatively less attention, suggesting that its constituent keywords represent relatively novel research directions. This indicates that research related to therapeutic strategies targeting Ferroptosis in cancer will constitute the core of future research endeavors. To go beyond static cluster analysis and more accurately reveal the dynamic evolutionary trajectory of research hotspots in this field, we further employed the Burst Detection function of CiteSpace software to conduct time-slice analysis on the keywords of literatures published from 2012 to 2025, and identified the top 20 burst terms with the highest intensity (**Figure 7C**, **Supplementary Figure 3**). Based on the start and end years of the burst of these keywords, we can clearly observe that the period from 2014 to 2021 belongs to the early foundation-laying and theoretical integration stage: the burst keywords during this period mainly focused on the core mechanisms and basic regulatory pathways of cell death. Examples include “death” (2014-2021), “calreticulin exposure” (2017-2021), “apoptotic cells” (2017-2019), and “nf kappa b” (NF-κB, 2018-2020). The burst periods of these keywords have now ended, indicating that the basic biological framework they represent has matured, integrated into the knowledge cornerstone of this field, and their academic popularity has gradually given way to research directions with greater translational potential. In contrast, the period from 2023 to 2026 is categorized as the recent emerging and frontier expansion stage: the current research frontiers exhibit three major characteristics, namely functionalization, systematization, and technological innovation. The consecutive bursts of “polarization” (2023-2024) and “macrophage polarization” (2024-2026) indicate that the research focus is shifting toward the functional phenotypic regulation of specific immune cells in the tumor immune microenvironment (20). The emergence of “gut microbiota” (2024-2026) as a systemic factor signifies that the research perspective in this field is expanding from the local tumor microenvironment to the distal organ-system crosstalk (21). The burst of “sonodynamic therapy” (2024-2026) reveals that innovative therapeutic strategies for non-pharmaceutical and physical induction of Ferroptosis are gaining widespread attention (22). Dynamic burst analysis demonstrates that the research focus of this field has shifted from the early mechanism discrimination and theoretical foundation-laying to the current precise functional regulation, complex system integration, and innovative technology application.

## 4 Discussion

### 4.1 General information related to publications

To our knowledge, this study is the first bibliometric analysis to systematically examine the Ferroptosis-immunity relationship. Furthermore, the methodological design of this study adheres to established guidelines for bibliometric research (23). Three databases—WOSCC, PubMed, and Scopus—were used to retrieve 3,436 relevant publications published between January 1, 2012, and August 31, 2025. These publications encompass contributions from 70 countries, 2,689 institutions, and 19,846 authors. The results reveal a marked upward trend in the number of publications in this field, with a dramatic surge occurring after 2020. This surge is driven primarily by the widespread reporting and validation of several landmark discoveries in the field: notably the in-depth characterization of key Ferroptosis regulatory pathways (e.g., GPX4 and System X_c_^⁻^), the identification of their synergistic interactions with cancer immunotherapies (e.g., immune checkpoint inhibitors), and the increased global focus on immune and cell death mechanisms spurred by the COVID-19 pandemic (20, 24). Given this momentum, a continued expansion in the number of publications in this area is anticipated.

Among the top 10 most productive countries in this research field, China and the United States stand out as the leading contributors in terms of publication output. This phenomenon stems in part from their substantial investment in scientific research, robust basic research capabilities, large research talent pools, strategic layout in the biomedical sector, and the presence of numerous top-tier research institutions and universities. Notably, despite China’s leading position in publication quantity, its average citations per article lag behind those of other top-producing countries, reflecting a relatively lower overall research quality. This highlights the need for Chinese researchers to prioritize improving the quality of their research outputs.

### 4.2 Research hotspots and trends

By analyzing the distribution of article counts across the top 10 research directions (**Table 3**), the field of Ferroptosis and immunology is anticipated to follow a multifaceted developmental trajectory. Notably, disciplines including Oncology, Biochemistry & Molecular Biology, Cell Biology, and Immunology ranked among the top tiers—underscoring their central role in driving advancements in Ferroptosis-related immunological disease research. As an iron-dependent form of programmed cell death, Ferroptosis exerts a critical influence on tumor initiation, progression, and response to therapeutic interventions. Accumulating studies have confirmed that Ferroptosis not only directly inhibits tumor growth by inducing cancer cell death but also enhances the body’s antitumor immune efficacy: it achieves this by releasing DAMPs that prime antitumor immune responses (25). Accordingly, Ferroptosis research in oncology will remain a focus of intense interest, particularly with respect to the detailed elucidation of its interaction mechanisms with the immune system and the translation of these insights into clinical practice.

**Table 3:** Top 10 research directions.

Rank	Research hotspots	Number of articles
1	Oncology	578
2	Biochemistry Molecular Biology	453
3	Cell Biology	431
4	Immunology	421
5	Pharmacology Pharmacy	299
6	Chemistry Multidisciplinary	287
7	Medicine Research Experimental	257
8	Nanoscience Nanotechnology	257
9	Multidisciplinary Sciences	210
10	Genetics Heredity	203

In the field of Cell Biology, Ferroptosis has emerged as a process involving intricate intracellular biological mechanisms. Accordingly, Cell Biology, Biochemistry Molecular Biology have established themselves as pivotal disciplines for investigating Ferroptosis, laying a foundational framework for advancing the understanding of its underlying mechanisms and facilitating translational applications.

Immunology emerges as a pivotal interdisciplinary domain in Ferroptosis research, having garnered sustained research attention and exhibited considerable promise in advancing related investigations. Looking ahead, future research directions are likely to encompass two key avenues: on the one hand, targeted regulation of Ferroptosis to augment the cytotoxic activity of immune cells against tumor cells; and on the other hand, harnessing Ferroptosis-elicited immune responses to refine the efficacy of cancer vaccines.

With the growing depth of insight into Ferroptosis mechanisms and ongoing advancements in associated technologies, Ferroptosis research is poised to yield novel insights and enable the development of therapeutic interventions for diverse diseases. Such research avenues not only bear substantial scientific significance but also harbor broad prospects for clinical translation. Leveraging multidisciplinary integration and collaborative innovation, studies focusing on Ferroptosis and immunity are expected to achieve further breakthroughs in the future, ultimately delivering substantial contributions to human health.

### 4.3 Hotspot keyword analysis

Our analysis revealed that “tumor immune microenvironment” (TME) emerges as a high-frequency keyword in recent years, as evidenced by both temporal distribution and keyword clustering analyses (**Supplementary Figure 2**). The TME is a multifaceted ecosystem encompassing tumor cells, immune cells, stromal cells, and other cellular components. These components do not merely coexist; they engage in intricate crosstalk that exerts profound regulatory effects on tumor growth and progression (26). Notably, Ferroptosis and the TME exhibit intricate interdependencies. During tumorigenesis and progression, rapidly proliferating tumor cells actively remodel the surrounding microenvironment, establishing a supportive niche that promotes their own survival while dampening anti-tumor immune responses. Concurrently, the TME profoundly modulates the Ferroptosis susceptibility of diverse cell populations within it, including tumor cells and immune cells (27). Ferroptosis induction directly dictates tumor cell fate, and its unique metabolic hallmarks (lipid peroxidation and iron dependency) position it as a critical hub connecting tumor biology and immune regulation. On one side, Ferroptosis induction can reshape tumor cell immunogenicity, thereby modulating immune cell function and responsiveness. On the other side, immune cells can actively regulate Ferroptosis in tumor cells via cytokine secretion or direct cell-cell contact (28). It is this multidirectional crosstalk that endows ferroptosis with a complex dual role in tumor immunity, namely pro-tumor and anti-tumor, and the final outcome is heavily dependent on specific cellular contexts and microenvironmental cues. Thus, a thorough dissection of the key molecular mechanisms underlying Ferroptosis, an elucidation of how it mediates immune responses, and a clarification of its dual roles within the TME are imperative for fully deciphering the biological significance of this triangular interplay (Ferroptosis-TME-immunity) and for developing novel Ferroptosis-targeted strategies in tumor immunotherapy.

### 4.4 Ferroptosis and tumor immunotherapy

Based on the analysis of research hotspots and keywords integrated with bibliometric results, we identified that the research direction linking Ferroptosis to oncology has emerged as a prominent topic in recent years. Specifically, oncology accounts for 16.8% of the total publications (ranking as the top research field), and the keyword “immunotherapy” appears 344 times. Herein, we focus on elaborating how the core mechanisms of Ferroptosis regulate tumor immune responses, thereby providing theoretical support for clinical translation.

Ferroptosis, a regulated form of cell death, has emerged as a focal point of interest in tumor research. Beyond functioning as a key intrinsic tumor-suppressive mechanism, it engages in intricate crosstalk with the tumor immune microenvironment to form a complex regulatory network. Notably, this dual role of Ferroptosis in tumor biology manifests in two distinct aspects:On one hand, Ferroptosis inducers (e.g., erastin, RSL3), clinically approved agents (e.g., sorafenib, sulfasalazine, statins, artemisinin), exposure to ionizing radiation, and specific cytokines (e.g., IFNγ, TGFβ1) can effectively induce Ferroptosis, thereby exerting tumor-suppressive effects. On the other hand, Ferroptosis may elicit inflammation-associated immunosuppression within the tumor microenvironment, which in turn promotes tumor progression (29). Thus, a comprehensive understanding of the multifaceted roles of Ferroptosis in tumorigenesis and progression is essential for translating Ferroptosis-targeted therapeutic strategies from preclinical research to clinical practice.

#### 4.4.1 Molecular mechanisms of ferroptosis

**Figure 8:**
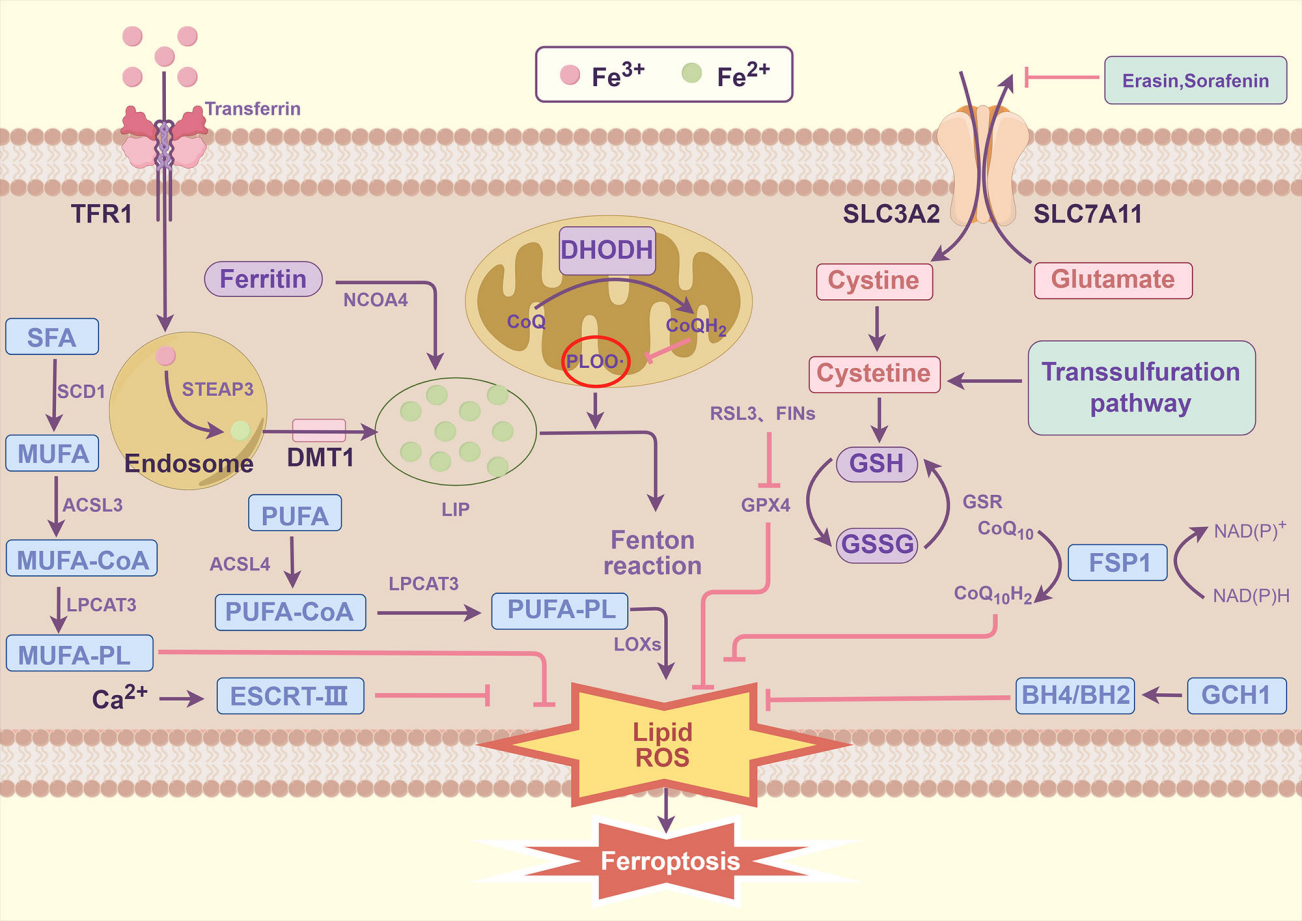
Key mechanisms of ferroptosis. ACSL3, acyl-CoA synthetase long-chain family member 3; ACSL4, acyl-CoA synthetase long-chain family member 4; BH4, tetrahydrobiopterin; CoQ10, coenzyme Q10; CoQ10H2, ubiquinol; DMT1, divalent metal transporter 1; FINs, ferroptosis-inducing agents; GSR, glutathione disulfide reductase; GSSG, glutathione disulfide; LIP, labile iron pool; LOX, lipoxygenase; LPCAT, lysophosphatidylcholine acyltransferase; NCOA4, nuclear receptor coactivator 4; RSL3, (1S,3R)-RSL3; SCD1 stearoyl-CoA desaturase 1; SFA, saturated fatty acid; SLC3A2, solute carrier family 3 member 2; SLC7A11, solute carrier family 7 member 11; STEAP3, six-transmembrane epithelial antigen protein 3; TFR1, transferrin receptor 1. By Figdraw.

Ferroptosis reflects a redox imbalance between its driving factors and defense systems. Here, we briefly outline its core mechanisms, placing particular emphasis on its driving and defense pathways. (As shown in **Figure 8**).

##### 4.4.1.1 Drivers of ferroptosis

###### 4.4.1.1.1 PUFA-PLs synthesis

The synthesis of polyunsaturated fatty acid phospholipids (PUFA-PLs) acts as a central driver of Ferroptosis. As a critical component of the cell membrane, PUFA-PLs are characterized by multiple double bonds that render them highly susceptible to oxidative stress and prone to lipid peroxidation. The PUFA-PL synthesis process initiates with the activation of free polyunsaturated fatty acids (PUFAs)—such as arachidonic acid (AA) or adrenic acid (AdA)—catalyzed by acyl-CoA synthase long-chain family member 4 (ACSL4), which generates PUFA-CoA. Next, lysophosphatidylcholine acyltransferase 3 (LPCAT3) mediates the transfer of PUFA-CoA to lysophospholipids, producing PUFA-PLs (e.g., phosphatidylethanolamine, PE) that are subsequently integrated into the cell membrane (30). During Ferroptosis, the expression of ACSL4 and LPCAT3 is upregulated, resulting in increased membrane PUFA-PL abundance and thereby supplying abundant substrates for lipid peroxidation (31, 32). Upon exposure to Ferroptosis inducers (e.g., erastin, RSL3), the double bonds in PUFA-PLs become susceptible to radical-induced oxidation, triggering lipid peroxidation chain reactions that produce lipid peroxides (e.g., lipid hydroperoxides) (33). These peroxides disrupt membrane integrity, enhance membrane permeability, and ultimately culminate in cell death. Notably, dietary PUFA intake also modulates Ferroptosis susceptibility; for example, high-PUFA diets exacerbate the pathological contribution of Ferroptosis in cancer and neurological disorders (34). Collectively, PUFA-PL synthesis serves as a central driver of Ferroptosis, primarily by supplying critical substrates for lipid peroxidation.

###### 4.4.1.1.2 Lipid peroxidation

Lipid peroxidation serves as the defining biochemical hallmark of Ferroptosis—a process characterized by radical-driven oxidation of PUFA-PLs, ultimately leading to the accumulation of toxic lipid peroxides. This biochemical cascade proceeds through three sequential stages: initiation, propagation, and termination. During initiation, hydroxyl radicals generated by reactive oxygen species (ROS) via the Fenton reaction attack the diallylic sites of PUFA-PLs, resulting in the formation of lipid radicals (35). In the propagation phase, lipid radicals react with molecular oxygen to produce lipid peroxyl radicals; these radicals then abstract hydrogen atoms from other lipid molecules, triggering an auto-replicative chain reaction (36). Termination, by contrast, depends on antioxidants that scavenge free radicals to generate stable non-radical products (36). In the context of Ferroptosis, accumulation of lipid peroxidation end-products—such as malondialdehyde (MDA) and 4-hydroxy-2-nonenal (4-HNE)—induces a reduction in membrane fluidity, an increase in permeability, and impairment of membrane protein function, ultimately culminating in cell membrane disintegration (37). Mitochondria, acting as primary targets of lipid peroxidation, display characteristic membrane crimping and mitochondrial membrane potential collapse, which together serve as key morphological hallmarks of Ferroptosis (6). Cumulative studies have confirmed that inhibiting LOX activity effectively abrogates the lipid peroxidation chain reaction, thereby alleviating Ferroptosis (38). Notably, lipid peroxidation exerts a dual role in Ferroptosis: it not only directly drives the cell death process but also exacerbates oxidative stress via positive feedback loops. Specifically, lipid peroxides further activate iron metabolic pathways, thereby establishing a self-amplifying death-signaling circuit (3).

###### 4.4.1.1.3 Iron metabolism and toxicity

Iron metabolism disorders and iron toxicity serve as primary drivers of Ferroptosis. Iron exists in two primary forms: ferrous iron (Fe^2+^) and ferric iron (Fe^3+^). Among these, Fe^2+^ generates ROS via the Fenton reaction, directly promoting lipid peroxidation. Cellular iron metabolism is a coordinated process and can be divided into three core components: iron uptake, storage, and export. For iron uptake, it is mediated by endocytosis through the transferrin receptor complex (TFRC); notably, Fe^3+^ is reduced to Fe^2+^ prior to its release into the cytoplasm via divalent metal transporter 1 (DMT1). Regarding iron storage, ferritin—composed of ferritin heavy chain (FTH1) and ferritin light chain (FTL)—sequesters iron to mitigate its toxic effects. For iron export, the membrane-bound iron transporter ferroportin facilitates this process (39). In the context of Ferroptosis, iron overload elevates the labile iron pool (LIP), thereby enhancing the Fenton reaction; this reaction further catalyses the peroxidation of PUFA-PLs. Iron toxicity additionally involves mitochondrial iron accumulation: here, the mitoferrin-dependent pathway and iron-sulphur cluster synthesis pathways collectively promote ROS generation (40). Under pathological scenarios (e.g., hemolysis or neurodegenerative diseases), elevated iron release further triggers Ferroptosis (41).

##### 4.4.1.2 Defenses of ferroptosis

###### 4.4.1.2.1 GPX4 antioxidant system

GPX4 serves as a central defensive mediator against Ferroptosis, primarily by reducing lipid peroxides to abrogate lipid peroxidation. As a selenoprotein, GPX4 employs glutathione (GSH) as a cofactor to catalyze the reduction of lipid hydroperoxides (LOOH) into their corresponding alcohols (LOH)—a reaction that directly interrupts the propagation of the lipid peroxidation chain reaction (42). Notably, GSH biosynthesis is tightly dependent on cystine uptake, which is mediated by the system X_c_^⁻^ transporter (composed of SLC7A11 and SLC3A2 subunits). Upon intracellular entry, cystine is reduced to cysteine—the rate-limiting substrate for GSH synthesis. In the context of Ferroptosis, distinct classes of inducers act on this regulatory axis: for instance, erastin inhibits system X_c_^⁻^ function, leading to GSH depletion and subsequent GPX4 inactivation; in contrast, direct GPX4 inhibitors such as RSL3 exert their effect via covalent binding to GPX4, thereby abrogating its enzymatic activity (43, 44). Functionally, deficiency or mutation of GPX4 promotes the accumulation of lipid peroxides, ultimately triggering Ferroptosis. Conversely, either overexpression of GPX4 or supplementation with selenium (a critical cofactor for GPX4 activity) bolsters cellular resistance against Ferroptosis (2).

###### 4.4.1.2.2 Radical-trapping antioxidant system

The free radical-scavenging antioxidant system serves as a key defense against Ferroptosis by disrupting the lipid peroxidation chain reaction. Endogenous lipophilic antioxidants (e.g., coenzyme Q10) terminate peroxidation propagation by donating hydrogen atoms to sequester lipid peroxy radicals (LOO•) and lipid radicals (L•), ultimately forming stable end products. Notably, the efficiency of this antioxidant system is tightly regulated by cellular metabolic status—for instance, NADPH levels directly modulate the regeneration of antioxidants, thereby influencing system function. Among such metabolic regulatory mechanisms, the NAD(P)H-FSP1-CoQ10 pathway acts as an independent parallel system: it mediates the reduction of ubiquinone to ubiquinol via FSP1, which in turn diminishes lipid radical formation, thereby suppressing lipid peroxidation and subsequent Ferroptosis (45). More recently, a study uncovered a mitochondria-localized defense system driven by dihydroorotic dehydrogenase (DHODH); this system compensates for GPX4 deficiency to detoxify mitochondrial lipid peroxidation, further expanding our understanding of Ferroptosis resistance (46). Beyond these pathways, the GCH1-BH4 axis also contributes to Ferroptosis regulation. GCH1 serves as the rate-limiting enzyme in tetrahydrobiopterin (BH4) synthesis, and BH4—along with its phosphorylated derivatives—exhibits robust radical-scavenging activity, enabling direct reduction of lipid radicals. A distinct feature of this axis is that GCH1 activation selectively generates antioxidant-enriched phospholipids, which deliver highly efficient localized protection within the membrane microenvironment. (47).

###### 4.4.1.2.3 MUFA-PLs synthesis

Monounsaturated fatty acid phospholipids (MUFA-PLs) bolster cellular resistance against Ferroptosis by remodeling membrane lipid composition. Notably, the absence of oxidizable diallyl moieties in their molecular structure endows MUFA-PLs—such as oleic acid-containing phospholipid species—with superior oxidative stability relative to PUFA-PLs (48). The synthesis of these protective lipids is initiated by stearoyl-CoA desaturase (SCD) in the endoplasmic reticulum, which catalyzes the desaturation of saturated fatty acids (e.g., stearic acid, C18:0) to generate MUFAs (e.g., oleic acid, C18:1). Subsequently, via a cascade of reactions involving acyl-CoA synthases (e.g., ACSL3) and lysophosphatidylcholine acyltransferases (e.g., LPCAT3), MUFAs are specifically incorporated into membrane phospholipids (49). Under ferroptotic stress, cells elevate the proportion of MUFA-PLs in membrane lipids either by upregulating SCD expression or exogenously supplementing MUFAs. This, in turn, competitively reduces the abundance of PUFA-PLs—the latter serving as critical substrates for lipid peroxidation (50).

###### 4.4.1.2.4 Membrane repair system

The membrane repair system functions as a late-stage defensive mechanism in Ferroptosis, delaying ferroptotic cell death through repairing membrane damage induced by lipid peroxidation. This system predominantly depends on the endosomal sorting complex required for transport (ESCRT) machinery, with particular reliance on its ESCRT-III subunits. Upon induction of membrane perforation or leakage by lipid peroxidation, ESCRT-III is recruited to the damaged sites, mediating membrane budding and scission to seal pores and eliminate damaged lipid domains (51, 52). In the context of Ferroptosis, oxidative stress drives ESCRT pathway activation; specifically, lipid peroxides can trigger calcium signaling cascades that facilitate the translocation of ESCRT components to the plasma membrane. Experimental evidence demonstrates that inhibition of ESCRT-III accelerates Ferroptosis progression, whereas its overexpression enhances cell survival during Ferroptosis (53). Collectively, by preserving membrane integrity, the membrane repair system affords cells a survival opportunity in Ferroptosis and thereby acts as the ultimate defensive line against ferroptotic cell death.

#### 4.4.2 Ferroptosis induction in tumor suppression

The ferroptotic pathway serves as an intrinsic tumor suppressor mechanism, mediating the anti-tumor effects of multiple tumor suppressor genes. Notably, key tumor suppressors—including p53 and BRCA1-associated protein 1 (BAP1)—have been shown to exert their tumor-suppressive functions by inducing, or at least partially triggering, Ferroptosis in tumor cells (54).

Beyond directly eliminating tumor cells, Ferroptosis further reshapes the tumor microenvironment toward a tumor-suppressive phenotype and sensitizes tumors to immunotherapeutic interventions (55). Notably, ferroptotic tumor cells release abundant damage-associated molecular patterns (DAMPs), including heat shock proteins, HMGB1, and nucleic acid fragments, which activate the antigen-presenting function of dendritic cells (DCs), thereby promoting DC maturation and initiating adaptive immune responses. (56, 57). Concurrently, Ferroptosis-driven lipid peroxidation products augment the exposure of tumor cell surface antigens, which improves T cell recognition efficiency and amplifies cytotoxic T lymphocyte (CTL)-mediated antitumor activity (58, 59). Specifically, Ferroptosis exhibits pronounced synergism with immune checkpoint inhibitors: it upregulates programmed death-ligand 1 (PD-L1) expression on tumor cell surfaces, while immune checkpoint inhibitors block the PD-1/PD-L1 pathway to not only relieve T cell suppression but also potentiate Ferroptosis-induced immunogenic cell death—thus establishing a “Ferroptosis-immune activation” positive feedback loop (60, 61). Currently, the combination therapy of Ferroptosis inducers and PD-1 inhibitors has advanced to clinical practice (62). Its preliminary efficacy not only provides clinical translational evidence for the “Ferroptosis-immunity” synergistic strategy but also validates the conclusion from this bibliometric analysis that “tumor immunity” represents a cutting-edge research hotspot. For instance, an ongoing Phase II clinical trial (NCT05294228) is evaluating the efficacy and safety of a glutathione peroxidase 4 (GPX4) inhibitor in combination with pembrolizumab (a PD-1 inhibitor) for the treatment of advanced solid tumors, which directly reflects the translational pathway from basic mechanism discovery to clinical intervention (63). Furthermore, another international multicenter trial (NCT04832269), collaborated by multiple research centers in China and the United States, is exploring the therapeutic effect of sorafenib (a known Ferroptosis inducer) combined with toripalimab (a PD-1 inhibitor) in the treatment of hepatocellular carcinoma, which also highlights the practical value of international cooperation in the field of Ferroptosis (64). Additionally, Ferroptosis modulates the polarization of tumor-associated macrophages (TAMs), driving their transition from immunosuppressive M2 to pro-inflammatory M1 phenotypes. This shift mitigates the immunosuppressive nature of the tumor microenvironment and further boosts immunotherapeutic efficacy (65).

Accumulating research evidence has established that canonical tumor suppressors, such as p53 and BAP1, exert their anti-tumor effects—at least in part—through inducing Ferroptosis in tumor cells; this mechanism is inherently coupled to synergistic crosstalk with the immune system (59). At the molecular regulatory level, p53 orchestrates Ferroptosis induction via a dual mechanism to mediate its tumor-suppressive functions: first, it directly represses the promoter activity of SLC7A11, thereby reducing cystine uptake and subsequent GSH biosynthesis; second, it impedes the serine transsulfuration pathway by inhibiting cystathionine β-synthase (CBS). Collectively, these two pathways synergistically impair the GPX4-dependent antioxidant defense system (66, 67). BAP1 encodes a deubiquitinating enzyme that catalyzes the deubiquitination of histone H2A. Specifically, BAP1 represses SLC7A11 expression by decreasing the occupancy of ubiquitinated histone H2A (H2Aub) at the SLC7A11 promoter, thus contributing to partial suppression of tumorigenesis through Ferroptosis induction (54). Importantly, BAP1-mediated Ferroptosis can elicit an immunogenic cell death response—via releasing DAMPs, activating DCs, and augmenting tumor antigen presentation (68). These research advances have laid a solid foundation for the combined therapeutic strategy of Ferroptosis and immunity. However, to advance this cutting-edge field toward maturity, a series of critical and actionable research questions remain to be addressed. For instance, the issue of synergistic resistance mechanisms: the preliminary clinical response rate of current combined therapy still has room for improvement. It is therefore necessary to thoroughly investigate how tumors evade the synergistic killing effect of Ferroptosis inducers and immune checkpoint inhibitors through intrinsic metabolic reprogramming (e.g., activation of alternative defense pathways such as FSP1-CoQ10) or utilization of the immune microenvironment (e.g., the protective role of tumor-associated macrophages, TAMs), so as to clarify the underlying resistance mechanisms (20, 69). Regarding the clinical validation and stratification of biomarkers, there is an urgent need to prospectively validate potential biomarkers identified in basic research (e.g., ACSL4 expression, lipid peroxidation levels, specific damage-associated molecular patterns (DAMPs) release profiles) in clinical cohorts. The goal is to establish a molecular classification system capable of predicting Ferroptosis sensitivity and the efficacy of combined immunotherapy, thereby achieving precise patient stratification (24, 70). For the development and delivery of novel inducers, the development of new compounds or nano-delivery systems that are highly effective, low-toxic, and capable of selectively inducing immunogenic Ferroptosis at tumor sites constitutes a core engineering challenge for improving the therapeutic window and safety (71). Clarifying these scientific questions will help focus the macro “research hotspot” into a clear translational pathway, thereby accelerating the advancement of Ferroptosis-based immunotherapy from proof-of-concept to widespread clinical benefit.

#### 4.4.3 Ferroptosis evasion in tumor progression

Despite the well-documented tumor-suppressive effects exerted via Ferroptosis, tumors still emerge and undergo uncontrolled proliferation. This observation implies that tumors have evolved Ferroptosis evasion mechanisms, which are rooted in Ferroptosis’ core regulatory drivers and cellular defense pathways (54). In the following, we briefly summarize the key mechanisms through which tumor cells acquire Ferroptosis resistance—ultimately enabling them to sustain tumor progression.

**Figure 9:**
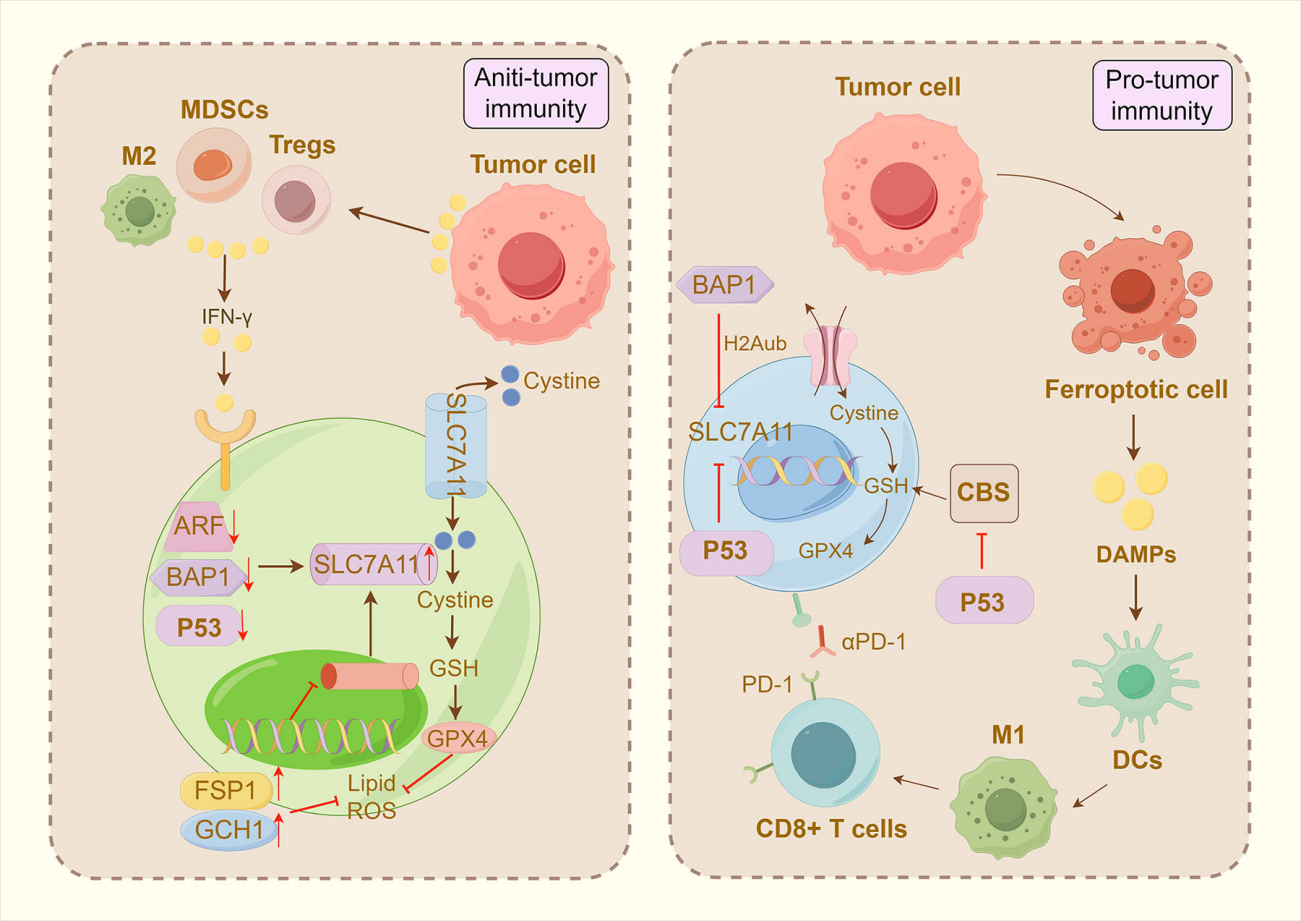
Tumor immune mechanisms of ferroptosis. ARF, p14ARF; BAP1, BRCA1-associated protein 1; CBS, cystathionine-β-synthase; DAMPs, damage-associated molecular patterns; DCs, dendritic cells; H2Aub, histone H2A monoubiquitination; IFN-γ, interferon-gamma; M1, M1-type macrophages; M2, M2-type macrophages; MDSCs, myeloid-derived suppressor cells; P53, P53 gene; PD-1, programmed death receptor 1; Tregs, regulatory T cells. By Figdraw.

Ferroptosis resistance serves as a critical strategy for tumor cells to evade programmed cell death and sustain their malignant proliferation. This resistance is characterized by enhanced antioxidant capacity, which acts as an adaptive response to elevated ROS levels driven by metabolic dysregulation and aberrant signaling. Upregulation of the SLC7A11/GSH/GPX4 axis—a central defensive axis against Ferroptosis—has emerged as a key evasion mechanism developed by tumor cells. Specifically, SLC7A11 overexpression has been documented in a broad spectrum of cancers and stands as one of the most extensively characterized mechanisms underlying tumor cell evasion of Ferroptosis (72, 73). For example, its expression is upregulated via the inactivation of tumor suppressor genes including TP53, BAP1, and ARF—an event that enables tumor cells to circumvent Ferroptosis and fuel their malignant growth (74). Accumulating evidence demonstrates that GSH levels are consistently elevated within the TME: on one hand, GSH functions as a pivotal cofactor for GPX4, thereby maintaining redox homeostasis by reducing lipid peroxides; on the other hand, it can deplete cysteinyl glutathione in the TME, impairing the metabolic function of immune cells (e.g., T cells) and in turn compromising antitumor immune responses (75, 76). As a core antioxidant enzyme governing Ferroptosis regulation, GPX4 is highly expressed in a wide range of tumor types. It not only inhibits Ferroptosis by catalyzing the reduction of lipid peroxides but also imparts anti-ferroptotic traits to neighboring immune cells through exosome-mediated intercellular communication, ultimately fostering the formation of an immunosuppressive TME (77). Notably, recent studies have uncovered that the Regulated Cell Death (RCD) pathway mediated by FSP1 and GCH1 plays a significant role in certain cancers. This pathway scavenges lipid radicals via non-GPX4-dependent mechanisms. It not only enhances tumor cells’ capacity to escape Ferroptosis and thereby promotes tumorigenesis, but also enables its metabolites (e.g., BH4) to modulate antitumor immunity by regulating dendritic cell maturation and T-cell activation (78). (As shown in **Figure 9**).

### 4.5 Ferroptosis and the tumor microenvironment

**Figure 10:**
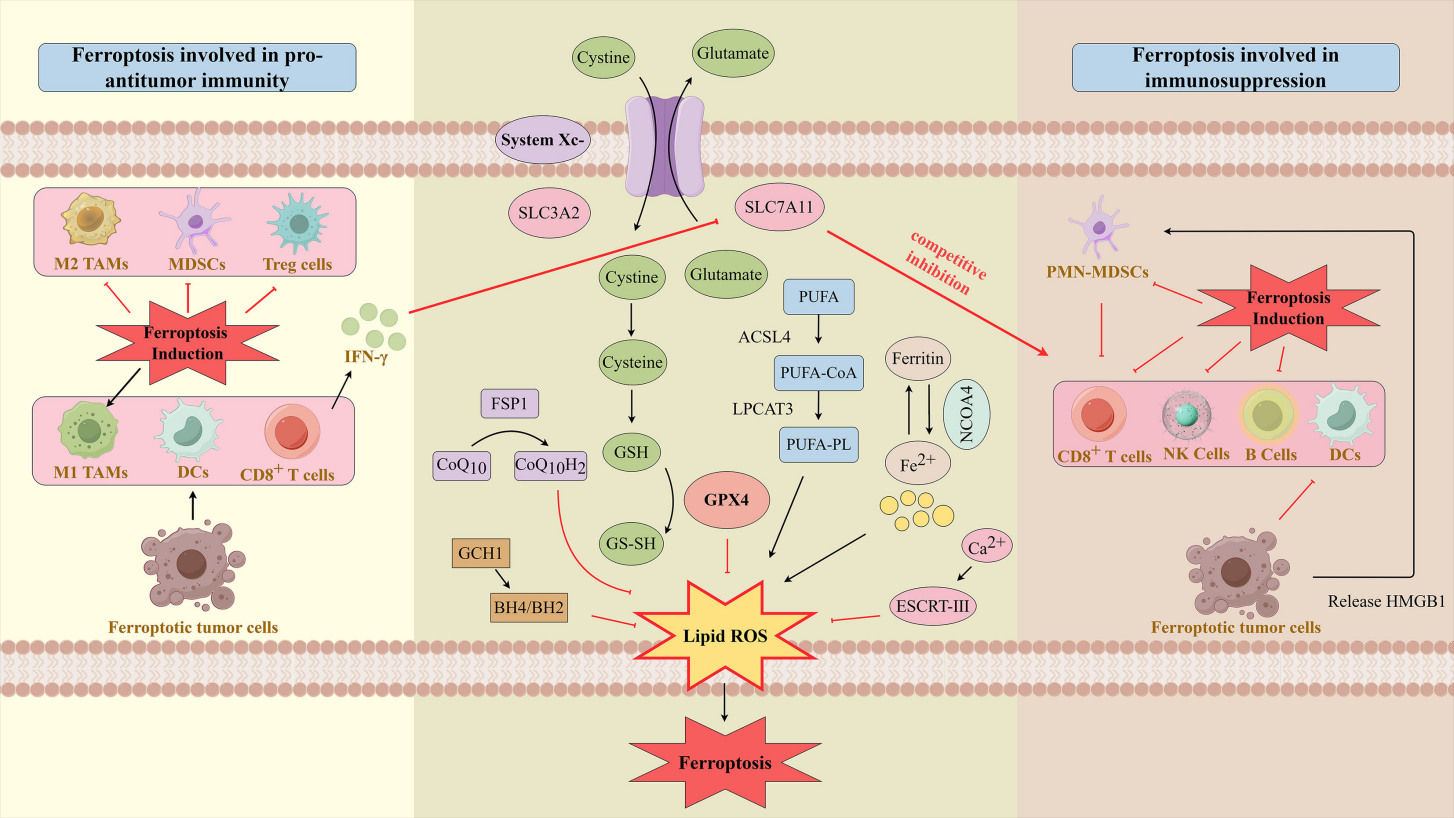
Bidirectional regulatory mechanisms of ferroptosis in tumor immunity. Left: Role of Ferroptosis in enhancing anti-tumor immunity. CD8^+^ T cells induce Ferroptosis in tumor cells via secreting interferon-γ (IFN-γ). Ferroptotic tumor cells release immunostimulatory signals, which promote dendritic cell (DC) maturation, activate M1-polarized macrophages, and enhance T cell infiltration and cytotoxicity in tumor tissues. Inducing Ferroptosis in immunosuppressive cells (myeloid-derived suppressor cells (MDSCs), regulatory T cells (Tregs), M2-polarized tumor-associated macrophages (TAMs)) impairs their immunosuppressive functions, thereby boosting anti-tumor immunity. Middle: Core Ferroptosis pathways. Key induction enzymes, including acyl-CoA synthetase long-chain family member 4 (ACSL4) and lysophosphatidylcholine acyltransferase 3 (LPCAT3), drive the synthesis of polyunsaturated fatty acid-containing phospholipids (PUFA-PLs), a prerequisite for lipid peroxide production. In contrast, the Ferroptosis defense system neutralizes lipid peroxides to block Ferroptosis and protect cells, mainly involving glutathione peroxidase 4-glutathione (GPX4-GSH), Ferroptosis suppressor protein 1-coenzyme QH_2_ (FSP1-CoQH_2_), and guanosine triphosphate cyclohydrolase 1-tetrahydrobiopterin (GCH1-BH4) pathways. Additionally, nuclear receptor coactivator 4 (NCOA4), a specific autophagic receptor, mediates ferritin degradation. It regulates Fenton reaction intensity by modulating intracellular free iron levels, thereby controlling lipid peroxide production and achieving bidirectional regulation of Ferroptosis. The cell membrane repair system also regulates Ferroptosis by scavenging lipid peroxides, repairing peroxidative membrane damage, or modulating iron metabolism-related pathways, ultimately reducing cell sensitivity to Ferroptosis. Right: Role of Ferroptosis in immunosuppression. Ferroptotic tumor cells release specific phospholipid peroxidation products that hinder DC maturation. Meanwhile, high mobility group box 1 (HMGB1) released by Ferroptotic tumor cells promotes the infiltration of immunosuppressive polymorphonuclear MDSCs (PMN-MDSCs) into tumor tissues. In some cases, Ferroptotic PMN-MDSCs inhibit CD8^+^ T cell activity. Inducing Ferroptosis in immunostimulatory cells (e.g., natural killer (NK) cells, B cells) typically impairs anti-tumor immunity. By Figdraw.

Within the TME, Ferroptosis serves as a pivotal mediator of bidirectional crosstalk between cancer cells and immune cells (As shown in **Figure 10**). Specifically, cancer cells undergoing Ferroptosis orchestrate antitumor immune responses through the release of context-specific signals; conversely, immune cells can in turn regulate the Ferroptosis susceptibility of cancer cells.

On the one hand, Ferroptosis serves as a critical catalyst for activating anti-tumor immunity. When cancer cells undergo Ferroptosis, they experience membrane rupture driven by lipid peroxidation, releasing DAMPs—notably HMGB1 and ATP. These signaling molecules are recognized by DCs within the TME. Upon sensing DAMPs via receptors such as Toll-like receptor 4 (TLR4), DCs exhibit a marked upregulation in the expression of surface co-stimulatory molecules (e.g., CD80, CD86) and major histocompatibility complex class II (MHC II) molecules. This upregulation promotes DC maturation, enhances their antigen-presenting capacity, and subsequently drives the efficient activation of cytotoxic T lymphocyte (CTL)-mediated anti-tumor responses (79, 80). Notably, oxidized lipids produced during Ferroptosis—e.g., certain hydroxy fatty acid derivatives—can also act as signaling molecules to directly or indirectly regulate immune cell function. Consequently, Ferroptosis in tumor cells induced by radiotherapy, chemotherapy, or agents targeting GPX4/System X_c_^⁻^ not only exerts direct cytotoxic effects on cancer cells but also reverses the immunosuppressive phenotype of the TME, thereby augmenting the therapeutic efficacy of immune checkpoint inhibitors (e.g., anti-PD-1 antibodies) (81). Importantly, this regulatory axis is not unidirectional: immune cells within the TME can actively exploit Ferroptosis as a weapon to target cancer cells. Activated CD8+ T cells are central to this process: they secrete IFN-γ, which downregulates the expression of SLC7A11—a core component of the System X_c_^⁻^ transporter on the cancer cell membrane. This downregulation directly impairs cystine uptake by cancer cells, reduces GSH biosynthesis, and inactivates GPX4 activity, thereby significantly increasing cancer cell susceptibility to Ferroptosis (82). This mechanism underscores how adaptive immune responses directly target metabolic vulnerabilities in cancer cells, mediating tumor cell clearance through Ferroptosis induction—a pathway that is critical for tumor elimination by the immune system.

Conversely, the inherent complexity of the TME underpins the double-edged role of Ferroptosis. Under specific contextual conditions, Ferroptosis may either impair immune cells directly or elicit immunosuppressive effects—two outcomes that counteract anti-tumor immunity. For instance, TAMs, particularly M2-polarized TAMs (a phenotype with strong immunosuppressive activity), can protect cancer cells from Ferroptosis. This protective effect is mediated either by secreting metabolites like lactate or through direct cell-cell contact (65). Conversely, certain immunosuppressive cells (e.g., regulatory T cells, Tregs) also exhibit sensitivity to Ferroptosis. Selective induction of Ferroptosis in Tregs can abrogate their suppressive effects on effector T cells, thereby reshaping the immune landscape of the TME. Notably, another dimension of this duality lies in the differential sensitivity of immunosuppressive cells: Tregs, a key mediator of TME immunosuppression, display inherent sensitivity to Ferroptosis. Thus, selective induction of Ferroptosis in Tregs holds potential for mitigating TME immunosuppression and restoring anti-tumor immune responses. Of greater concern, the abundant lipid peroxides and their end products (e.g., 4-HNE) generated during Ferroptosis possess high chemical reactivity. These reactive species can exert direct toxicity on effector immune cells—such as infiltrating CD8+ T cells—inducing functional exhaustion or outright cell death, thereby compromising anti-tumor immunity (54). Beyond immune cell interactions, intercellular crosstalk within the TME further modulates Ferroptosis to favor tumor survival. For example, cancer-associated fibroblasts (CAFs) can support tumor cell resistance to Ferroptosis by two distinct mechanisms: secreting pro-survival signaling molecules or supplying essential metabolites like cysteine (83). Additionally, tumor cells may transfer antioxidant molecules (e.g., GPX4) via exosomes, a process that confers Ferroptosis resistance across the entire tumor cell population rather than individual cells (84). (As shown in **Figure 10**).

### 4.6 Limitations

This bibliometric study systematically delineates the fundamental research landscape, hotspots, and evolutionary trends in Ferroptosis and immunity via a visual analytical approach. The findings provide a holistic reference framework for researchers currently engaged in or interested in this field. Notably, this study is not without limitations. First, to enable robust software-driven analysis, data were retrieved exclusively from three databases (WoSCC, PubMed, and Scopus), while other prominent academic platforms such as Google Scholar and Embase were excluded from the analysis. Second, the literature search for this study was conducted up to August 31, 2025. However, papers published between 2024 and 2025 may not have accrued a sufficient number of citations, which could introduce biases into the keyword trend analysis and cutting-edge research identification. Third, limiting the inclusion criteria to English-language publications potentially undermined the comprehensiveness of the literature search. These limitations warrant careful consideration when interpreting the study’s findings. Nevertheless, this work provides valuable insights into the core research areas and emerging frontiers of the Ferroptosis-immunity field.

## 5 Conclusion

This study presents the first systematic mapping of the research landscape in the Ferroptosis-immunity field by synthesizing data from three major databases: WoSCC, PubMed, and Scopus. China is the leading contributing region, hosting the most productive authors and academic institutions in this field. We strongly advocate for enhanced collaboration and knowledge exchange across countries, institutions, and research teams to advance field development.

In recent years, key research hotspots have converged on Ferroptosis within the fields of Oncology, Biochemistry Molecular Biology, Cell Biology, and Immunology. Among these, research on Ferroptosis and the TME has emerged as a dominant focus in cancer therapeutics. Future research should prioritize further elucidation of Ferroptosis mechanisms in tumor immunotherapy, as well as the exploration of potential therapeutic targets for Ferroptosis-targeting agents in cancer therapy.

In summary, this study offers a comprehensive overview of research hotspots and developmental trends in the field, providing robust data support for subsequent investigations into Ferroptosis-immunity mechanisms and the development of related therapeutic strategies. We anticipate the generation of more high-quality research outcomes and the establishment of collaborative networks in the future, which will collectively drive the in-depth advancement of Ferroptosis-immunity research.

## Data Availability

Publicly available datasets were analyzed in this study. This data can be found here: <b>https://webofscience.clarivate.cn/wos/woscc/summary/63399ae2-954a-4a36-aa47-8bc5220b8107-0186dc2acd/relevance/1https://pubmed.ncbi.nlm.nih.gov/?term=%28ferroptosis%5BTitle%2FAbstract%5D%29+AND+%28immunity%5BTitle%2FAbstract%5D+OR+immune%5BTitle%2FAbstract%5D%29&sort=&filter=dates.2012%2F1%2F1-2025%2F8%2F31&filter=dates.2012%2F1%2F1-2025%2F8%2F31https://www.scopus.com/results/results.uri?st1=ferroptosis&st2=immunity+OR+immune&yearFrom=2014&yearTo=2025&s=%28TITLE-ABS-KEY%28ferroptosis%29+AND+TITLE-ABS-KEY%28immunity+OR+immune%29%29&limit=10&origin=resultslist&sort=plf-f&src=s&sot=b&sdt=cl&sessionSearchId=cecebe150363474c899beccb877d0b5d&cluster=scosubtype%2C%22bk%22%2Cf%2C%22tb%22%2Cf%2C%22cp%22%2Cf%2C%22cr%22%2Cf%2C%22er%22%2Cf%2C%22le%22%2Cf%2C%22ch%22%2Cf%2C%22sh%22%2Cf%2C%22no%22%2Cf%2C%22ed%22%2Cf%2Bscolang%2C%22Chinese%22%2Cf%2C%22Russian%22%2Cf%2C%22Ukrainian%22%2Cf%2C%22catalan%22%2Cf%2C%22Japanese%22%2Cf%2C%22German%22%2Cf%2C%22French%22%2Cf%2Bscosubjabbr%2C%22PHYS%22%2Cf%2C%22COMP%22%2Cf%2C%22MATH%22%2Cf%2C%22PSYC%22%2Cf%2C%22SOCI%22%2Cf%2C%22EART%22%2Cf</b>.
