# Supplementary material for: Ferroptosis and immunity: a bibliometric analysis of research hotspots and frontiers (2012-2025)
Source: Front Immunol. 2026 Jan 12;16:1739210. doi: 10.3389/fimmu.2025.1739210 (PMC12833263; doi:10.3389/fimmu.2025.1739210)
Supplement: Supplementary file 1 [file DataSheet1.docx]

Supplementary Material

# Supplementary Figures and Tables

- 1. **Supplementary Figures**

**
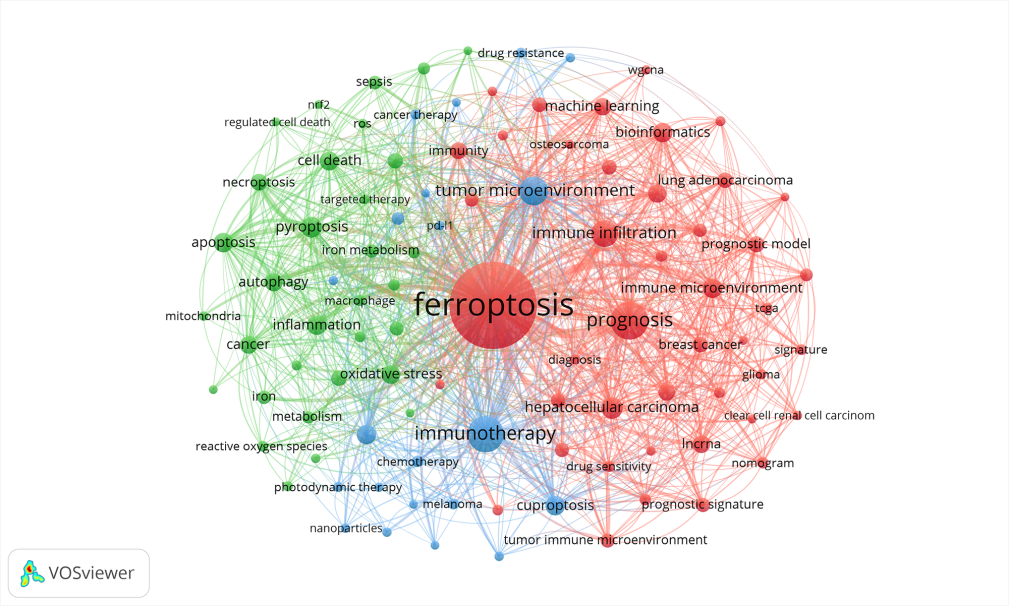
**

**Supplementary Figure 1.** Literature keyword contribution chart. The size of the circle represents the frequency of occurrence.


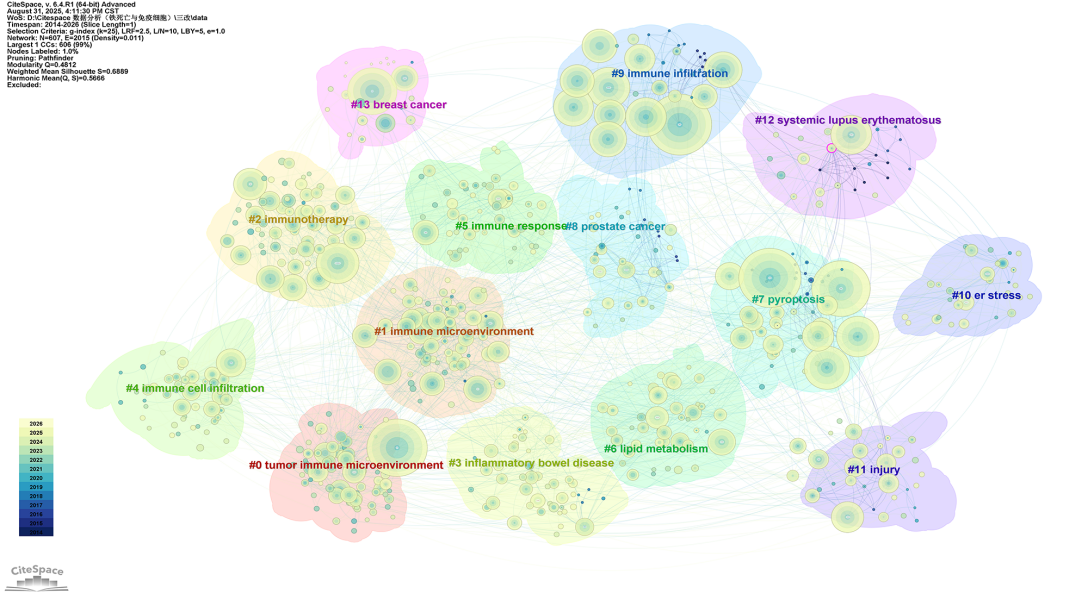


**B**

**A**


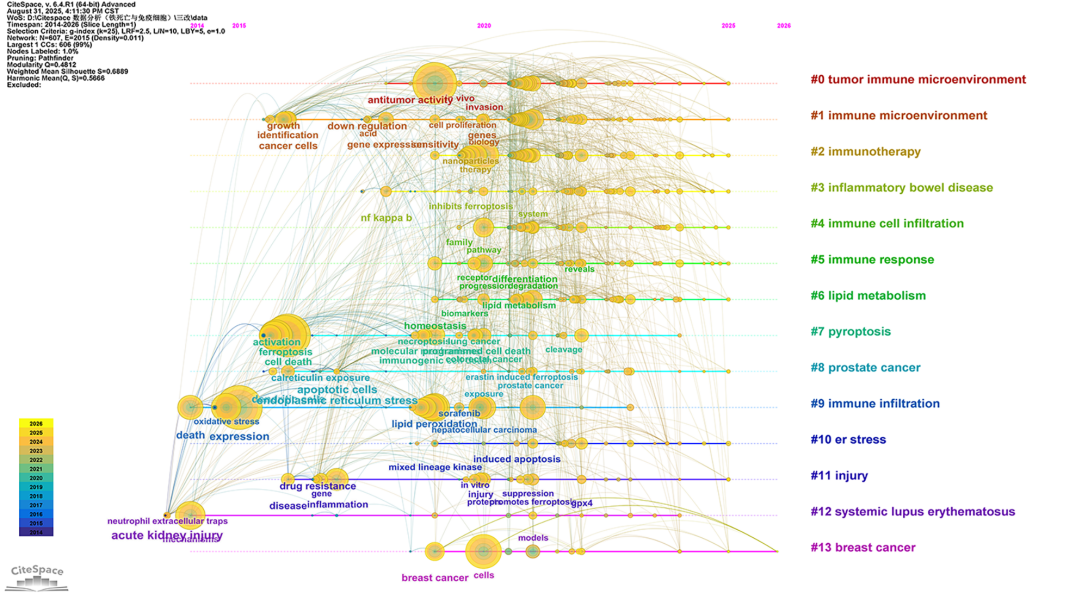


**Supplementary Figure 2.** Keyword cluster analysis. **A.** Keyword cluster graph. The size of nodes indicates the frequency of occurrences of the keywords, and the lines between the nodes represent their co-occurrence in the same publication. **B.** Keyword clustering timeline chart. Keywords concentrate on the time period from 2015 to 2023.


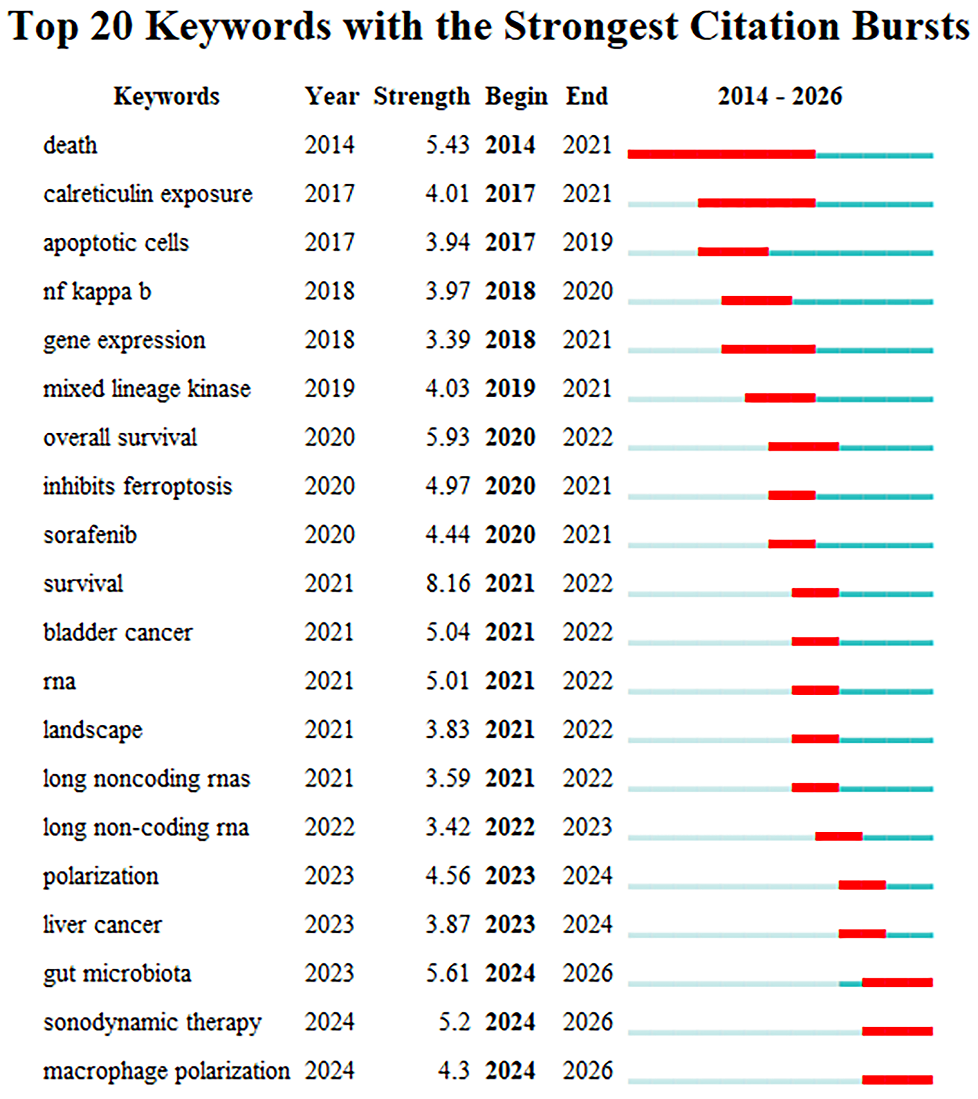


**Supplementary Figure 3.** The top 20 keywords with the strongest citation bursts. The “Year” column denotes the publication year when a keyword first appeared. “Strength” reflects the intensity of the citation burst. “Begin” and “End” specify the start and end years of the burst period, respectively. In the timeline diagram on the right, the light blue bars represent periods when the keyword was present but not experiencing a burst, while the red bars indicate the active burst periods.

- 1. **Supplementary Tables**

**Table 1** Literature keyword frequency TOP30.

| Serial number | Occurrences | Total link strength | Keyword | Serial number | Occurrences | Total link strength | | Keyword |
| --- | --- | --- | --- | --- | --- | --- | --- | --- |
| 1 | 1967 | 3521 | ferroptosis | 16 | 90 | 247 | autophagy | |
| 2 | 362 | 936 | prognosis | 17 | 88 | 208 | biomarker | |
| 3 | 344 | 786 | immunotherapy | 18 | 84 | 196 | machine learning | |
| 4 | 214 | 566 | tumor microenvironment | 19 | 83 | 173 | cancer | |
| 5 | 191 | 424 | immune infiltration | 20 | 79 | 214 | lncrna | |
| 6 | 123 | 291 | hepatocellular carcinoma | 21 | 77 | 189 | immunity | |
| 7 | 114 | 341 | pyroptosis | 22 | 77 | 253 | necroptosis | |
| 8 | 102 | 221 | bioinformatics | 23 | 75 | 190 | immune | |
| 9 | 102 | 179 | inflammation | 24 | 69 | 174 | prognostic model | |
| 10 | 101 | 216 | immunogenic cell death | 25 | 66 | 136 | lipid peroxidation | |
| 11 | 100 | 297 | apoptosis | 26 | 66 | 176 | lung adenocarcinoma | |
| 12 | 100 | 273 | cuproptosis | 27 | 65 | 170 | breast cancer | |
| 13 | 100 | 247 | immune microenvironment | 28 | 63 | 167 | programmed cell death | |
| 14 | 92 | 204 | cell death | 29 | 58 | 118 | bioinformatics analysis | |
| 15 | 92 | 161 | oxidative stress | 30 | 58 | 133 | biomarkers | |

Total Link Strength: A parameter that quantifies the aggregate strength ofall connections between a node and other nodes. An increased number ofconnections and greater strength contribute to a higher total link strength.
